# The preventive care of medication-related osteonecrosis of the jaw (MRONJ): a position paper by Italian experts for dental hygienists

**DOI:** 10.1007/s00520-022-06940-8

**Published:** 2022-03-16

**Authors:** Rodolfo Mauceri, Rita Coniglio, Antonia Abbinante, Paola Carcieri, Domenico Tomassi, Vera Panzarella, Olga Di Fede, Francesco Bertoldo, Vittorio Fusco, Alberto Bedogni, Giuseppina Campisi

**Affiliations:** 1 Department of Surgical, Oncological and Oral Sciences (Di.Chir.On.S.), University of Palermo, Via L. Giuffrè 5, 90127 Palermo, PA, Italy; 2 Department of Biomedical and Dental Sciences, Morphological and Functional Images, University of Messina, Messina, Italy; 3 Department of Dental Surgery, Faculty of Dental Surgery, University of Malta, Msida, Malta; 4 Italian Dental Hygienists Association - AIDI, Aosta, Italy; 5 Department of Interdisciplinary Medicine, Complex Operating Unit of Odontostomatology, Aldo Moro University of Bari, Bari, Italy; 6 Department of Surgical Sciences, CIR-Dental School, Oral Medicine Section, University of Turin, Turin, Italy; 7 CIR-Dental School, Oral Prevention and Community Dentistry, University of Turin, Turin, Italy; 8 Catholic University of Rome, Rome, Italy; 9 National Union of Dental Hygienists – UNID, Rome, Italy; 10 Department of Medicine, University of Verona, Verona, Italy; 11 Oncology Unit, Azienda Ospedaliera Di Alessandria SS, Antonio e Biagio E Cesare Arrigo, Alessandria, Italy; 12 Regional Center for Prevention, Diagnosis and Treatment of Medication and Radiation-Related Bone Diseases of the Head and Neck, University of Padua, Padua, Italy

**Keywords:** Osteonecrosis of the jaw, MRONJ, Dental hygienists, Prevention, Risk factors, Periodontal screening score

## Abstract

**Purpose:**

The prevention and early diagnosis of medication-related osteonecrosis of the jaw (MRONJ) is fundamental to reducing the incidence and progression of MRONJ. Many in the field believe that dental hygienists should play an integral role in primary and secondary MRONJ prevention. However, to date, very few publications in the literature have proposed standardised MRONJ protocols, which are dedicated to dental hygienists. The aim of this study was to provide guidance to the health care providers managing MRONJ.

**Methods:**

The expert opinion in this study was developed by dental hygienists from the main Italian technical-scientific associations (Italian Dental Hygienists Association, AIDI and National Union of Dental Hygienists, UNID) and authors of the latest Italian recommendations regarding MRONJ from the field of dentistry and maxillofacial surgery.

**Results:**

The oral care protocol outlined in this position paper is focused on the role of dental hygienist in patients at risk or affected by MRONJ, and it regards 3 main issues: primary prevention, secondary prevention and supporting the treatment of MRONJ. Each issue contains easy-to-apply indications and procedures, as described by the authors, regarding the role of the dental hygienist.

**Conclusion:**

Referring to the main issues under consideration (primary prevention, secondary prevention and the treatment of MRONJ), a clinical examination of periodontal tissue is critical in preventing MRONJ. It is the opinion of the authors of this study that the application of a periodontal screening score is fundamental in defining personalised strategies for patients at risk of MRONJ. By means of these basic procedures, a protocol for assisting the health care provider and the presentation of a practical approach for patients at risk or affected by MRONJ are described in this study.

**Supplementary Information:**

The online version contains supplementary material available at 10.1007/s00520-022-06940-8.

## Introduction

Osteonecrosis of the jaw (ONJ) can be defined as “an adverse reaction, which is characterised by the progressive destruction and necrosis of the mandibular and/or maxillary bone, in subjects exposed to treatment with drugs with an established increased risk of disease, in the absence of previous radiation treatment” [1, 2].

The disease mostly affects cancer patients and patients with osteometabolic diseases, who are exposed to drugs with antiresorptive activity (AR; e.g. bisphosphonates, denosumab) and/or drugs with anti-angiogenic action (AA; e.g. bevacizumab) [2–10]. Indeed, ONJ has been associated with several drugs; thus, the term medication-related osteonecrosis of the jaw (MRONJ) is frequently used in the literature and clinical practice [3, 5, 11].

MRONJ can greatly affect the quality of the life of patients; whilst its aetiopathogenesis is still unclear, its incidence and progression can be greatly reduced through primary and secondary prevention [12–17].

MRONJ is a multi-professional issue, and all the healthcare professionals involved play an important role to play in order to prevent and manage MRONJ [18]. Although the role of the hygienist is fundamental to the development of preventive strategies relating to MRONJ, only a few papers in the international literature have addressed the work of the dental hygienist[19–22]. This position paper is the result of the work of a Board of experts, in the light of the following: new findings, the publication in 2020 of the new Italian recommendations regarding MRONJ and the best practice shared in the 2021 *ONJ Update* Conference (www.onjupdate.it) [2, 3, 6, 23–31].

This report includes the contribution of dental hygienists who belong to the main technical-scientific associations—*Italian Dental Hygienists Association* (AIDI) and *National Union of Dental Hygienists* (UNID) and authors of the above-mentioned Italian recommendations. This expert opinion has focused on the role of dental hygienist in patients at risk or affected by MRONJ by referring to 3 main issues: primary prevention, secondary prevention and the treatment of MRONJ. Herein, the authors have described easy-to-apply indications and procedures relating to each issue in order to reduce the risk of MRONJ onset, to make an early diagnosis and to support its treatment [2]. However, in order to facilitate the reading of this paper, the following is considered a prerequisite: a knowledge of MRONJ risk factors, a classification of patients at risk of MRONJ and diagnostic criteria and staging of the disease. Knowledge regarding these topics is described in Appendix 1 and in the latest Italian recommendations regarding MRONJ [2]. Similarly, an up-to-date working knowledge of periodontal disease will be pertinent in Appendix 2.

1. **MRONJ primary prevention**

An appropriate preventive approach is that most effective strategy for protecting the oral health of a patient, who will be taking, is taking or has taken drugs associated with a risk of MRONJ [13–17, 32]. Specifically, the aim of primary prevention is the control of local risk factors, which are related to the MRONJ pathology prior to and during the commencement of drug therapy (and even after its cessation).

The aim of primary prevention is to maintain and/or restore periodically the patient’s state of dental-periodontal health in order to achieve two objectives:

- To perform non-invasive dental procedures in order to reduce the possibility of developing or progressing of oral risk factors, such as infectious events
- To perform invasive procedures (e.g. dental extraction by dentist), where indicated for teeth with a poor prognosis

The additional aim of primary prevention is appropriate counselling, through which the patient is informed of the risk of MRONJ and made aware of its possible clinical manifestations, thereby facilitating early diagnosis. Contemporaneously, the dental hygienist should inform patients of the beneficial effects of AR and/or AA medications. This would encourage medication compliance, as recommended by the treating physicians and reduce the risks of bone fracture (e.g. hip fracture) and the related complications (e.g. death) [33].

All physiological and/or pathological conditions which directly or indirectly compromise an optimal oral health status, especially at the dento-periodontal level, render the jaw more susceptible to infection [2, 3, 5, 26]. The dental hygienist can play a pivotal role in the prevention of MRONJ: They should be responsible for implementing professional oral hygiene protocols to achieve the following objectives:

- Control oral risk factors
- Maintain/restore oral dento-periodontal health
- Maintain/improve patient compliance and cooperation

Primary prevention procedures in oncological patients (ONC) should always begin prior to the commencement of a given drug therapy, in accordance with ministerial recommendations and continue during and after drug therapy with ONJ-related drugs [34]. Primary prevention procedures in the osteometabolic patient (OST) are recommended within 6 months from the commencing of treatment with ONJ-related drugs (Table 1) [2].

**Table 1:** Dental hygienist’s intervention timing with patients at risk of MRONJ

Patient type	Groups	Timing of the dental hygienist’s intervention
Oncological (ONC)	Pharmacological pre-treatment (R_0_)	ALWAYS PRIOR to treatment
	Undergoing pharmacological treatment (R_+,_ R_++_)	ASAP, if not examined pre-treatment Periodic follow-up (every 4 months)
Osteometabolic (OST) oncological with CTIBL*	Pharmacological pre-treatment (R_0_)	Within 6 months of commencing treatment
	Undergoing pharmacological treatment (R_x_)	ASAP, if not examined pre-treatment Periodic follow-up (every 6 months)

*NB* Oncological patients are classified on the basis of *diverse risk* (R), subdivided into 3 subgroups: ONC-R0 (if ONJ-related drug administration planned but not yet commenced); ONC-R + (if ONJ-related drug therapy commenced); and ONC-R +  + (if concomitant or subsequent drug therapy with anti-angiogenic activity and/or with local and/or systemic risk factors). Patients with osteometabolic pathology at risk of MRONJ can be divided into two subgroups: OST-R0 (subjects with no risk) and Ost-Rx (subjects with potentially increased risk compared to OST-R0, although not definable as “x”) (Appendix 1). *Cancer patients receiving hormone therapy with CTIBL: cancer treatment induced bone loss (iatrogenic bone loss)

## Primary prevention in patients who are prescribed ONJ-related drugs

Primary prevention measures for MRONJ will be introduced during the first meeting on the basis of the evaluation of patient’s oral health status (Table 2). Reviewing the patient’s clinical and radiological records will provide the first indication as to the patient’s health [35]. Clinical assessments begin with the screening procedures of periodontal tissue. The board suggests the use of periodontal screening and recording (PSR) [36] to facilitate the simultaneous identification of MRONJ local risk factors. This method is indicated to efficiently differentiate clinical diagnoses and to plan the detection of any periodontal damage. Indeed, PSR is an effective procedure, which can be applied to any patient (Table 3) [36, 37]. Clinicians may use either a WHO periodontal probe or a University of North Carolina colour-coded probe.

**Table 2:** Sequence of primary prevention intervention performed by the dental hygienist

1	Patient interview, evaluation of clinical and radiological documentation
2	Decontamination of the bacterial count with chlorhexidine-based mouthwash (to be repeated before any treatment and/or evaluative action) [38]
3	Clinical evaluation and possible update in the clinical notes with: • Screening of periodontal/peri-implant tissue and teeth (e.g. PSR) • Screening for other local risk factors (e.g. dentures) • Screening of oral mucosal lesions • Svaluation of salivary flow rate (at rest)
4	Rationale and explanation of the use of home oral hygiene tools and paying prompt attention to the signs and symptoms of MRONJ
5	Professional oral hygiene (supra-subgingival debridement and/or deplaquing)
6	Application of remineralising agents
7	Counselling regarding lifestyle habits (e.g. smoking and/or alcohol consumption) (where necessary)
8	Planning personalised periodic follow-up appointments

**Table 3:** PSR codes [36]

Code 0: Colour-coded reference mark is completely visible in the deepest sulcus or pocket of the sextant. No calculus or defective margins on restorations are present. Gingival tissues are healthy with no bleeding evident on gentle probing
Code 1: Colour-coded reference mark is completely visible in the deepest sulcus or pocket of the sextant. No calculus or defective margins on restorations are present. Bleeding is present on probing
Code 2: Colour-coded reference mark is completely visible in the deepest sulcus or pocket of the sextant. Supragingival or subgingival calculus and/or defective margins are detected
Code 3: Colour-coded reference mark is partially visible in the deepest sulcus or pocket of the sextant. This code indicates a probing depth between 3.5 and 5.5 mm
Code 4: Colour-coded reference mark is not visible in the deepest sulcus or pocket in the sextant. This code indicates a probing depth of greater than 5.5 mm
An asterisk * will be appended to the code of a sextant, exhibiting any of the following abnormalities: furcation involvement; mobility; mucogingival problems; recession extending into the coloured area of the probe

After an assessment of oral health, a further assessment of all local risk factors (potential or present) is recommended (e.g. tooth decay). If the patient uses removable dental prostheses, the suitability, stability and quality of maintenance of these prostheses should be assessed [2, 39]. Where fixed prostheses are used, the following must be evaluated: the marginal seal (e.g. protruding edges, the presence of secondary caries) and the patient’s ability to maintain at-home oral hygiene. The oral mucous membranes should also be assessed to identify any lesions, which should be described in the medical notes and recorded photographically. Where mucosal lesions and/or opportunistic infections are present, the dental hygienist should refer the patient to an oral medicine specialist, a specialist in oral surgery or to a MRONJ referral centre for diagnosis and treatment.

It should be highlighted that numerous pathologies which can influence the development of periodontitis, such as diabetes mellitus, are contemporaneously classified with the comorbidities facilitating the development of MRONJ and they must be investigated during an examination [2, 40–44]. Moreover, it should be underlined that MRONJ patients are usually frail patients. For example, as a result of the chemo-/radiology therapies of cancer patients, additional complications involving the oral tissue may occur, and these may well adversely affect the patient’s general condition and oral health homeostasis [45]. These include the following: mucositis, xerostomia and/or hyposalivation, dysphagia and opportunistic infections, all of which may compromise oral physiological functions [34]. These adverse effects can also have repercussions on the maintenance of oral health homeostasis [46–48].

The dental hygienist should discuss the rationale and explanation of the use of home oral hygiene tools with the patient. Much of the treatment success in preventive measures lies with the dental hygienist's ability to engage with the patient to effect behavioural changes in controlling modifiable local and systemic risk factors. Only the motivated and informed patient will be able to perform effective oral hygiene practices at home [3, 4, 25, 49]. These indications will be defined according to: the general clinical picture, the morphology of the oral cavity and teeth, the gingival phenotype, the patient’s cooperation and manual aptitude. The choice and use of at-home oral care devices should be made on a patient-by-patient basis, and the following needs to be taken into account: learning time and compliance regarding their use, when to use oral care devices, associated techniques for use and the tools to use [36]. Electric tooth brushing is recommended as the primary means of plaque control, with soft filaments being preferred. Where the gingivae are inflamed, interdental cleaning, preferably with interdental brushes, should be made available to the patient. The clinician may also suggest other interdental cleaning devices/methods (e.g. flossing, the use of a single tufted brush), the use of which is personalised for each patient [50]. Mineral content toothpaste is preferred, and a specific toothpaste chosen according to the needs of each patient is recommended (e.g. fluorinated, desensitising, probiotic-containing). As an adjuvant to mechanical plaque control, chemical control is by means of antibacterial mouthwashes/gel, depending on the various treatment or maintenance stages. The efficacy of such substances has been widely addressed in the literature, and it would appear that chlorhexidine-based antiseptic mouthwashes assist in maintaining a healthy oral microbiome [35, 38].

If the patient uses fixed prostheses, it will be necessary to suggest that the patient use dental floss with a floss threader or threader-tip floss, which permits insertion between the prosthetic elements and/or a low-powered water brush, because food residues and plaque can easily accumulate under the prostheses, which are often difficult to remove by brushing alone. Where the patient has dental implants, the instruments for home cleaning must not contain metal parts which could be abrasive, thereby encouraging the accumulation of plaque and calculus. With removable prostheses, daily cleansing with specialised brushes and products is recommended. Where the patient has implant-supported overdenture, it should be explained to the patient to clean the implant/anchor screw (in addition to the prosthesis).

It is strongly recommended that the dental hygienist encourages the patient’s awareness and involvement in self-assessment to promptly report signs or symptoms of MRONJ to the clinician (e.g. sudden teeth loosening, abscesses) (Appendix 1). The patient will be a protagonist of early diagnosis in providing information to the clinician [2].

Finally, the dental hygienist will be responsible for suggesting counselling techniques regarding lifestyle choices (e.g. smoking).

After these primary prevention interventions, the next step will be professional oral hygiene (Table 2). Professional mechanical plaque removal includes interventions, the aim of which is to remove plaque and calculus. This is in addition to any plaque retention factors (e.g. incongruous restorations), which could compromise oral hygiene. During professional treatment, the choice of dental instruments and applied techniques should always prioritise lesser invasive intervention regarding oral tissue. It is essential to use effective dental instruments which are also appropriate to the treatment site, whether they are manual or mechanical instruments (e.g. sonic/ultrasonic). To date, ultrasound debridement has proved to be the most effective and least traumatic technique for the removal of calculus concretions in non-surgical periodontal treatment [50]. Instruments with the latest-generation technology (e.g. air polishing), utilising reduced particle size powders, are of assistance in removing biofilm (deplaquing) [51, 52]; deplaquing is also indicated when rehabilitating prosthetic implants. A plaque detector can be used prior to deplaquing to facilitate plaque identification and removal (preferably with biphasic or triphasic polishing). In cases of periodontal disease, the professional hygiene session should include subgingival debridement, using minimally invasive instruments (thin tip inserts and/or mini or micro mini-five curettes), where necessary under local anaesthetic. Where comorbidities are present, it will be the responsibility of the dentist to assess the requirement for antibiotics (e.g. to mitigate the risk of bacterial endocarditis) [53]. Basing their decision on the patient’s health, it will be the decision of the dental hygienist and dentist to assess treatment type (that is, the quadrant or full-mouth approach).

**Fig. 1:**
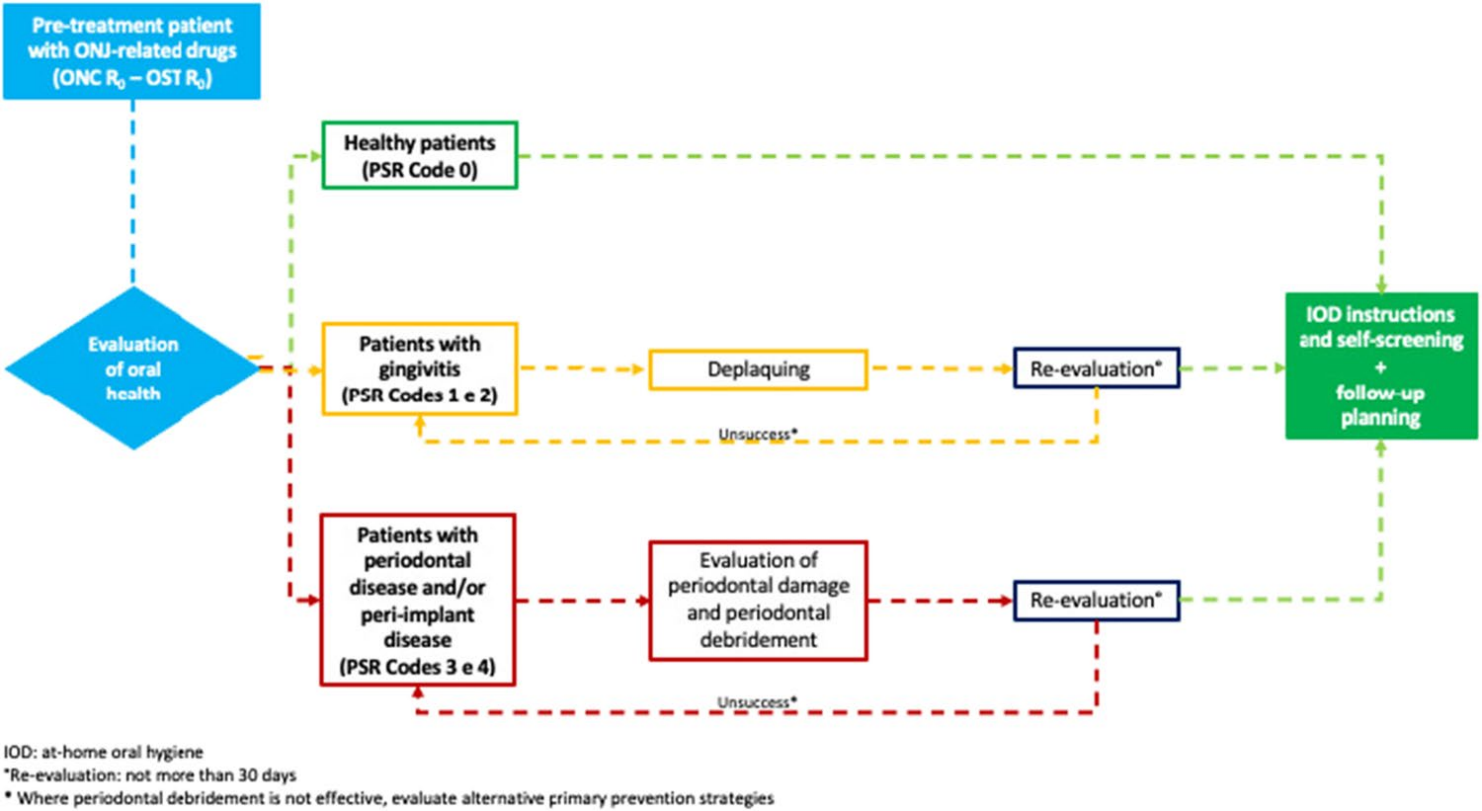
Flow chart pathway for primary prevention pre-treatment with ONJ-related medication: ONC R_0_ and OST R_0_ patients

Patients in pre-treatment undergoing professional hygiene treatment should be reassessed within 30 days. The envisaged clinical picture is periodontal tissue healing; the patient will be included in medium- to long-term follow-up treatment (ONC at 4 months, OST at 6 months) (Fig. 1) [2]. On the basis of measured PSR indices, periodic and personalised follow-up appointments may be scheduled. In the interests of clarity and on the basis of the PSR Code, the periodontal conditions for which a patient may be a candidate for therapy are detailed below.

### Healthy patient (PSR Code 0)

Where clinical signs of inflammation are absent, there is no presence of calculus or plaque retention factors; the patient should be considered periodontally healthy (Code 0) [54–56]. However, the patient should be encouraged to maintain oral health and self-assessment to promptly report any early signs or symptoms of MRONJ. Thereafter, the oncological patient will be included in a 4-month follow-up, whilst the next check-up for the osteometabolic patient will be scheduled at 6 months (Fig. 1).

### Patients with gingivitis and/or mucositis (PSR Codes 1 and 2)

PSR Codes 1 and 2 describe gingivitis (without periodontitis) and/or the presence of plaque retention factors [54–56]. After employing the measures described in Table 2, the patient will be re-evaluated within the next 30 days. This re-evaluation will assess the following: tissue response to treatment, the effectiveness of the patient’s plaque control techniques and, where necessary, the possibility of correcting inappropriate practices. If the measures implemented are effective, standardised follow-up appointment will be arranged (ONC 4 months, OST 6 months) [2].

In patients with dental implants, it should be considered that implant sites with clinical signs of mucositis may be present (e.g. bleeding on probing (BOP), mild erythema, swelling and/or suppuration). The patient will be treated with the same objectives for the treatment of gingivitis, but with the appropriate equipment for implant treatment [57]. If the clinical picture of inflammation persists at re-evaluation, the dental hygienist will identify its causes, attempt to reduce the inflammation and remind the patient of the importance of preventive measures, thereby encouraging patient compliance (Fig. 1).

### Patients with periodontitis and/or peri-implantitis (PSR Codes 3 and 4)

For a patient who, on initial assessment, has been identified with Codes 3 and 4, the dental hygienist should proceed with a thorough assessment of periodontal damage (periodontal charting) [54–56]. In this subgroup of patients, treatment can be provided by conventional periodontal debridement techniques. Where there is tooth mobility, the continuous stress on the deep tissue can cause discomfort and lead the patient to avoid the physiological use of the compromised teeth and to reduce cleaning practices. Once the degree of mobility and the prognosis for the teeth have been assessed, splinting the mobile teeth can be considered.

In addition to periodontitis, peri-implantitis is also a significant risk factor for MRONJ [26, 58–60]. It should be treated with the same objectives regarding the treatment of periodontitis, using appropriate equipment. A re-evaluation of the periodontitis/peri-implantitis patient should be performed within 30 days of treatment being concluded. The desired clinical outcome is the healing of the periodontal/peri-implant tissue, which will be followed by scheduling the patient’s follow-up appointment (ONC 4 months, OST 6 months) (Fig. 1). Where there is persistent inflammation, consideration should be given to repeating non-surgical treatment, with additional counselling to the patient regarding the need for complying with at-home oral hygiene protocols. Alternatively, prevention strategies, which are compatible with the patient's primary pathology, can be evaluated with the dentist (e.g. dental avulsion).

*The Board believes that clinical-radiological* ***evaluation of dental-periodontal health*** *in patients with an* ***oncological pathology*** *should* ***ALWAYS*** *be performed* ***PRIOR*** *to commencing ONJ-related medication. Any periodontal tissue disease should be treated promptly in order to reduce the risk of MRONJ. In patients with* ***osteometabolic disease*** *, the initial dental evaluation is not mandatory prior to commencing with AR drugs, but its performance is recommended* ***WITHIN THE FIRST 6 MONTHS*** *of AR drug therapy.*

## Primary prevention in patients receiving ONJ-related drugs

If the patient presents at the dental clinic during treatment with ONJ-related drugs (or after the conclusion of treatment with AR), typically the oral health specialist should record the medical history data and perform a clinical-radiological examination. Subsequently, they will evaluate the presence or absence of local risk factors and the risk of MRONJ (Appendix 1) [2]. As is the case with patients who are pre-treated with ONJ-related drugs, the sequence of actions by the dental hygienist should be repeated (Table 2). Furthermore, it is important to remember that it is also important to apply secondary preventive measures in patients who have taken ONJ-related drugs. Periodically, the dentist should repeat radiographic examinations during check-up appointments (e.g. intraoral X-ray, every 12 months). On the basis of the measured PSR indices, targeted preventive measures can be performed, and periodic and personalised follow-up appointments planned. The periodontal conditions of patients, who are candidates for therapy with ONJ-related drugs, will be obtained by referring to the PSR code.

### Healthy patients (PSR Code 0)

**Fig. 2:**
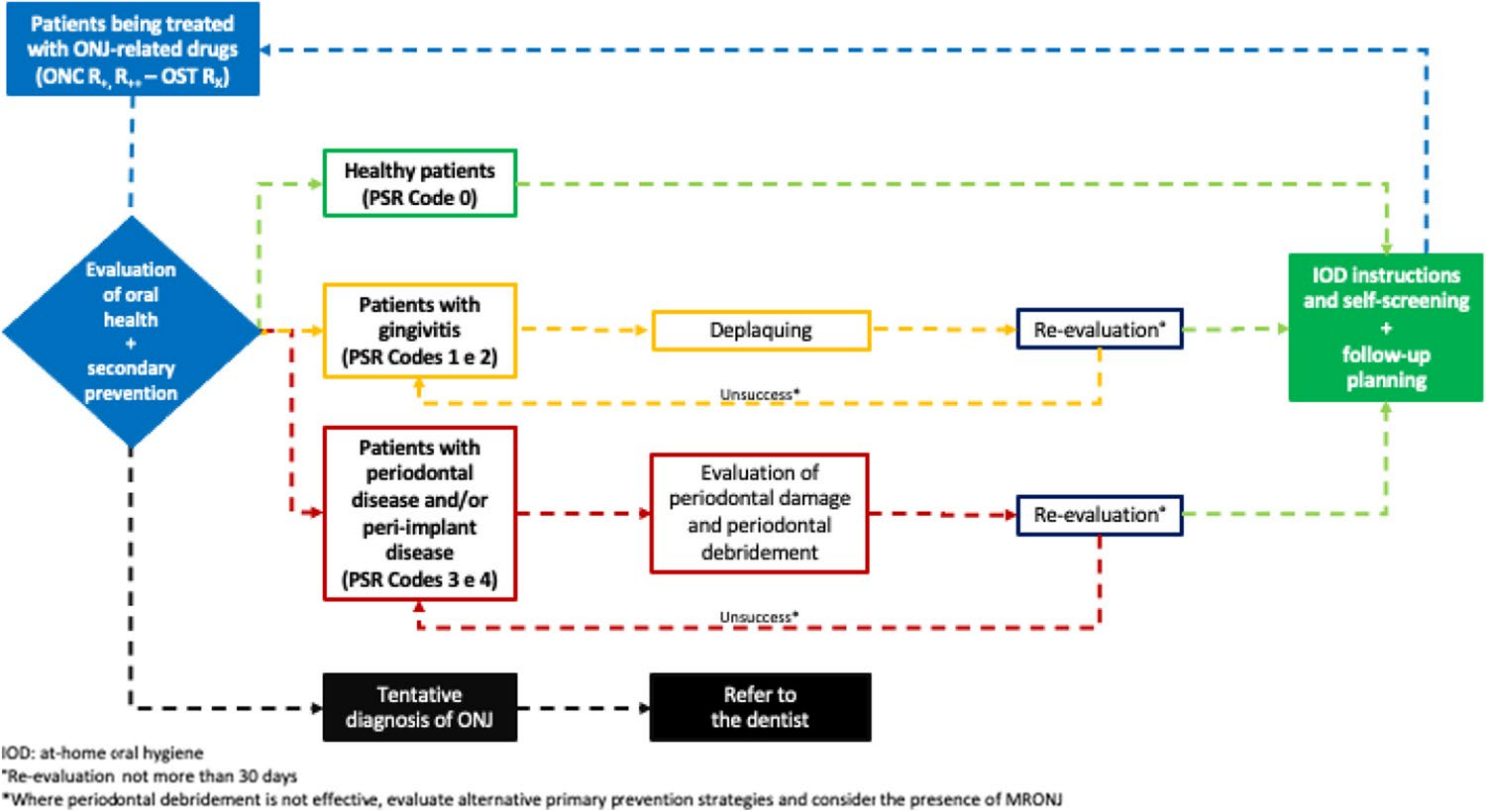
Flowchart of primary prevention pathway during treatment with ONJ-related medication: ONC R_+_, R_++_ and OST R_x_ patients

Where gingival inflammation is absent, the patient with healthy periodontal tissue should be included in a periodic follow-up (ONC 4 months, OST 6 months) (Fig. 2) [2, 49].

### Patients with gingivitis and/or mucositis (PSR Codes 1 and 2)

Prompt action must be taken to eliminate inflammation to prevent it from developing into periodontal disease. If plaque and calculus are present, they should, therefore, be removed using mechanical and/or manual instruments with the least invasive techniques. Thereafter, the patient should be re-evaluated within 30 days, and, where the preventive measures have been effective, the patient can be placed in medium- to long-term follow-up (ONC 4 months, OST 6 months) [2, 49]. If gingivitis persists, the patient should attend subsequent oral hygiene sessions and be reminded of the necessity of maintaining effective at-home oral hygiene with the aim of encouraging patient compliance (Fig. 2). As previously described, the clinician should treat the patient with mucositis with the same objectives as those for treating gingivitis and appropriate equipment [57].

### Patients with periodontal disease and/or peri-implantitis (PSR Codes 3 and 4)

Where there are clinical signs of periodontitis and/or peri-implantitis, the dental hygienist will widen their initial assessment by investigating tissue damage by means of periodontal probing (e.g. CAL) in order to ascertain the extent of periodontal tissue loss. Treatment can be provided by conventional periodontal debridement techniques; a re-evaluation of the patient’s health after non-surgical periodontal treatment should be performed within 30 days after treatment has been concluded. The envisaged clinical picture is the healing of the periodontal tissue, which will include the patient in a standardised follow-up pathway (ONC 4 months, OST 6 months) (Fig. 2) [2, 49]. The persistence of inflammatory foci will require repeated treatment, also reminding the patient of the requirement to comply with maintaining effective at-home oral hygiene. Contemporaneously, alternative primary prevention strategies should be evaluated with the dentist.

Moreover, as previously described, it is important to remember that numerous pathologies and adverse effects of cancer treatments may affect the development of periodontitis and/or peri-implantitis as well as facilitating the development of MRONJ (e.g. diabetes mellitus) [40–44, 46–48]. Attention should also be paid to any early clinical-radiological signs of the early stages of MRONJ [2].

*The Board considers it important to commence preventive care protocols in patients at risk of MRONJ. The use of an oral care pathway is indispensable; its typology will be determined on the basis of the medical data collected during the first dental examination and an individual patient’s risk factors. At each subsequent check-up, the medical history must always be updated, especially information relating to the use of ONJ-related medication.*

2) MRONJ secondary prevention

The aim of MRONJ secondary prevention is early diagnosis (i.e. the recognition of all those clinical/radiological signs and/or symptoms), which can be associated with an early stage MRONJ[4, 49].

Patients may be considered to have MRONJ if all the following characteristics are present [2, 4, 25, 61, 62]:

- Current or previous treatment with antiresorptive (AR) or antiangiogenic agents (AA)
- Clinical-radiological findings of progressive bone destruction
- No history of radiation therapy to the jaws or presence of cancer lesions (e.g. oral squamous cell carcinoma) or metastatic disease to the jaws

Of importance, it was recently suggested that patients taking AR medications and presenting with signs of bone necrosis in previously radiated jaws should be regarded as true MRONJ cases if they received less than 40 GY radiation dose at the necrosis site [63]. This suggested radiation dose cut-off is not included, at present, in any published Expert Panel Recommendation and will require further confirmation.

**Fig. 3:**
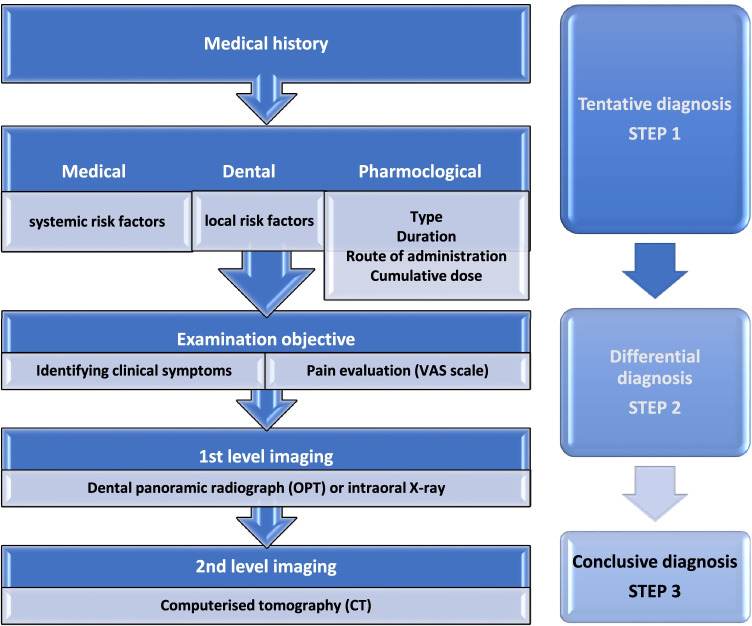
Diagnostic work-up of MRONJ ( modified from SICMF-SIPMO) [2]

A diagnostic work-up permits the clinician to make a tentative diagnosis (Step 1), via the means of a differential diagnosis (Step 2) in order to reduce the time required for a final diagnosis by the physician (Step 3) (Fig.3) [2].

### Step 1

The possibility of MRONJ should be raised whenever a patient being treated (current or previous) with high-risk medication presents oral symptoms and symptoms compatible with MRONJ. The presence of such symptoms should prompt radiological investigation to confirm or exclude any doubt (Appendix 1) [64].

### Step 2

The differential diagnosis must consider all those pathological conditions of the oral cavity presenting clinical and radiological symptoms, which may overlap with the initial phases of MRONJ, or which may be a precipitating factor for MRONJ. At this stage, the dental hygienist will play an important role in the differential diagnosis between periodontal disease, endo-periotic abscesses and the early stages of MRONJ and in referring the patient for further evaluation to the dentist or to specialised centres for the diagnosis of MRONJ.

### Step 3

Patients should be referred to specialised centres for the treatment of MRONJ (e.g. outpatient clinics of oral medicine, oral surgery, maxillofacial surgery) where more specific investigations and a final reassessment will be performed.

*The Board considers it essential for the dental hygienist to evaluate the secondary preventive care protocols in patients at risk of MRONJ. Indeed, early diagnosis of MRONJ is usually associated with early therapeutic strategies, and improved outcomes.*

3) The role of the dental hygienist in the treatment of MRONJ

The role of the dental hygienist is central to medical and surgical approaches as maintaining optimal oral hygiene is the minimum prerequisite for the therapeutic success of MRONJ (Table 4). A comprehensive periodontal/peri-implant health assessment (e.g. PSR) should be performed and recorded in the medical notes. Preliminary preparation and effective professional control of oral bacterial plaque can

- Alleviate, where present, painful gingival symptoms experienced by the patient, thereby mitigating these symptoms from affecting their quality of life.
- Control superinfection by inhibiting lesion progression.

**Table 4:** Main treatment strategies for MRONJ ( modified from SICMF-SIPMO) [2]

Medical treatment	Antiseptic treatment
	Antibiotic treatment
	Pain-relief treatment
	Discontinuing current drug therapy
	Teriparatide
	Bio-stimulation: - Ozone therapy - Laser therapy - Hyperbaric oxygen therapy
Surgical treatment	Surface osteoplasty
	Dento-alveolar curettage
	Sequestrectomy
	Resective surgery (marginal or segmental)

Based on the patient’s periodontal/peri-implant health and general clinical picture, minimally invasive professional oral hygiene measures should be performed with the aim of re-establishing the maintenance of optimal oral hygiene before MRONJ surgical therapy. In addition, chlorhexidine-based mouthwashes (at different concentrations, based on need) may be indicated.

If it is decided to treat MRONJ conservatively, the dental hygienist (on the advice of the dentist) could play a central role in all bio-stimulation procedures. These may be performed using ozone-generating instruments or laser therapy [2, 65–72]. The application of ozone therapy deploys various aids and methods (e.g. insufflations). Ozone acts by stimulating and/or preserving the endogenous antioxidant system, activating blood circulation, stimulating biological reactions (promoting bone sequestration) and exerting a bactericidal action and reducing pain [65–68].

Bio-stimulation by laser therapy (e.g. low level laser therapy, LLLT) would seem to be effective in increasing the organic bone matrix in the proximity of the lesion, in stimulating the growth of blood and lymph vessels inside and outside the gum line and in reducing pain and possibly also the size of adjacent bone exposure. And this is in addition to its characteristics of being a safe, minimally invasive and well-tolerated technique [68–72]. Many authors have reported clinical success in the treatment of MRONJ with LLLT, deploying different wavelengths and different parameters [73, 74].

It should be noted that the main objectives in the treatment of MRONJ are to control infection, to slow the disease’s progression and to promote tissue healing; however, the optimal MRONJ management is still a matter of controversy [2, 75]. Indeed, there are several articles on the various modalities of MRONJ therapy, although there is a lack of sufficient scientific evidence to define the gold standard treatment.

*The Board believes that the latest scientific evidence points to the need for the prompt surgical treatment of MRONJ. The role of the dental hygienist is central in this regard in: preparing the patient for surgery, maintaining the patient's oral health and, if available, applying conservative adjuvant healing techniques.*

## Conclusion

To our knowledge, this is the first position paper in the literature describing the best practice of dental hygienists and MRONJ prevention procedures. Preventive approaches may be considered the most appropriate strategy for diminishing the risk of MRONJ in patients who are candidates for ONJ-related drugs, during and after treatment [12–17]. These actions usually form part of a multidisciplinary approach, involving professional figures, who are responsible for the prevention of oral disease: dentists and dental hygienists. The authors of this paper wish to draw attention to the role of dental hygienist in preventing oral health disease and in safeguarding the patient’s health. The dental hygienist has appropriate training which facilitates the identification and checking for the risk of MRONJ [76].

Furthermore, whilst it is evident that periodontal pathology and MRONJ have substantially different aetiologies, they regard the same tissue. Thus, the former can facilitate the development of the latter. Periodontitis is an important risk factor for MRONJ; its eradication and maintenance is a priority intervention with patients at risk of MRONJ [2]*.* Another factor enhancing the dental hygienist’s role in a tentative diagnosis of MRONJ is the frequency with which the dental hygienist comes into contact with patients. The dental hygienist intervenes in a preventive manner via periodic oral hygiene check-ups, during which the patient’s general condition is re-evaluated, and this includes extra- and intra-oral examinations. Thus, any changes in the patient’s health will be detected, in addition to any suspicious clinical symptoms of MRONJ being identified at an early stage.

In conclusion, the professional nature of the dental hygienist can lead the practitioner to activate each and every clinical and communication strategy to the benefit of the patient’s oral health. By means of their expert opinion, the authors of this position paper aspire to impart knowledge relating to MRONJ to the professional community, with the aim of reducing the risk of this pathology, thereby protecting the oral health of our patients.

## Supplementary Information

Below is the link to the electronic supplementary material.

Supplementary file1 (DOCX 1160 KB)

Supplementary file2 (DOCX 63 KB)

## Data Availability

Not applicable.

## Declarations

### Disclaimer

This expert opinion is based on a narrative review of the existing data and the clinical observations of an expert task force, which is composed of professionals experienced in the diagnosis and management of MRONJ. The expert opinion is informational in nature; the authors caution that the strategies described in this position paper might not be suitable for every, or any, purpose or application. Additionally, this expert opinion cannot substitute the individual judgment brought to each clinical situation by a patient’s healthcare providers. As with all clinical materials, this position paper should be used with the clear understanding that continued research and practice could result in revision or updated recommendations.
