# Supplementary material for: The preventive care of medication-related osteonecrosis of the jaw (MRONJ): a position paper by Italian experts for dental hygienists
Source: Support Care Cancer. 2022 Mar 16;30(8):6429–40. doi: 10.1007/s00520-022-06940-8 (PMC9213300; doi:10.1007/s00520-022-06940-8)
Supplement: Supplementary file 1 — Supplementary file1 (DOCX 1160 KB) [file 520_2022_6940_MOESM1_ESM.docx]

**The preventive care of Medication-related Osteonecrosis of the Jaw (MRONJ): a Position Paper by Italian experts for dental hygienists**

**Authors**

Mauceri Rodolfo*^1,2,3^, Coniglio Rita^1^, Abbinante Antonia^4^, Carcieri Paola^5^, Tomassi Domenico^6^, Panzarella Vera^1^, Di Fede Olga^1^, Bertoldo Francesco^7^, Fusco Vittorio^8^, Bedogni Alberto^9^, Campisi Giuseppina^1^.

**Affiliations**

^1^ Department of Surgical, Oncological and Oral Sciences (Di.Chir.On.S.), University of Palermo, Palermo, Italy

^2^ Department of Biomedical and Dental Sciences, Morphological and Functional Images, University of Messina, Messina, Italy

^3^ Department of Dental Surgery, Faculty of Dental Surgery, University of Malta, Msida, Malta

^4^ Italian Dental Hygienists Association - AIDI, Aosta, Italy

^5^ Department of Surgical Sciences, Oral Medicine Section, CIR-Dental School, University of Turin, Turin, Italy - Oral prevention and community dentistry, CIR-Dental School, University of Turin, Turin, Italy

^6^ Catholic University of Rome, Rome, Italy - National Union of Dental Hygienists – UNID, Rome, Italy

^7^ Department of Medicine, University of Verona, Verona, Italy.

^8^ Oncology Unit, Azienda Ospedaliera di Alessandria SS, Antonio e Biagio e Cesare Arrigo, Alessandria, Italy

^9^ Regional Center for Prevention, Diagnosis and Treatment of Medication and Radiation-Related Bone Diseases of the Head and Neck, University of Padua, Padua, Italy.

*Corresponding Author

Rodolfo Mauceri,

Sector of Oral Medicine, Department of Surgical, Oncological, and Oral Sciences (Di.Chir.On.S),

University of Palermo, Palermo, Italy

Via L. Giuffrè 5, 90127

Palermo (PA), Italy

rodolfo.mauceri@unipa.it

**Appendix 1**

**Risk factors for MRONJ**

One of the characteristics of MRONJ is its almost exclusive localisation in the maxillary bones. The possible causes of this phenomenon are not yet fully known but a number of reasons have to date been hypothesised, as follows:

- there is physiologically higher bone turnover in the maxillary bones than in the rest of the skeleton[1, 2]

- the terminal vascularisation of the mandible[3]

- the presence of a thin muco-periosteal lining (for protecting the underlying bone tissue), which is easily subject to trauma[2]

- the distinctive microflora/biofilm of the oral cavity[4] and

- a characteristic dento-alveolar interface, which may be predisposed to the exposure of the underlying bone tissue, where there is dento-periodontal disease (e.g. periapical lesions and abscesses, periodontal disease) or oral-dental surgery[5].

The risk factors which are currently considered to be associated with MRONJ can be divided into three groups (Table 1):

- drug-related risk factors

- systemic risk factors and

- local risk factors

It is essential to identify the factors distinguishing patients with high/low/indefinable risk of MRONJ.

***Drug-related risk factors***

There are mainly two classes of drugs implicated in the etiopathogenesis of MRONJ, with different activity regarding bone metabolism:

- drugs with **predominantly antiresorptive (AR) activity**: bisphosphonates (BP) and denosumab and
- drugs with **predominantly anti-angiogenic activity (AA)**: anti-vascular endothelial growth factor (VEGF)(e.g. bevacizumab), tyrosine kinase (TK) inhibitors (e.g. sunitinib) and mammalian Target of Rapamycin (mTOR) inhibitors (e.g. everolimus).

The bioavailability of these drugs, and thus their intrinsic potential for MRONJ-specific causation, varies with multiple factors, as detailed below.

*Molecule type*

BPs, in which the amine group is present (amino-bisphosphonates, N-BPs), have a remarkable affinity for bone. Of these drugs, zoledronate can be considered as the most potent N-BPs[6, 7]. An alteration in bone turnover is the primary effect of BPs, and it can result in a reduction in osteoclastic activity. Denosumab is also characterised by a prevalent antiresorptive action through the inhibition of the RANK-L (Receptor Activator of Nuclear factor-kB Ligand) complex and, thus, osteoclastic activity, with reduced bone remodelling and increased mineral density[8].

The forms of MRONJ, which are caused by AA drugs are quantitively smaller, but they are evidently increasing in number and in the presence of case reports; these forms are linked to new drugs, which are implicated in the development of MRONJ[9–13]. They share the probable, multi-factorial aetiopathogenesis, involving the infected part and the immune system. In addition, the mechanisms regulating bone repair are strongly related to neo-angiogenesis and, therefore, the suppression of pro-angiogenetic factors contributes to a reduction in bone remodelling[14]. This activity would also seem to justify the increased risk of developing MRONJ in patients receiving combined treatment (AA+AR) or treatment with multiple drugs with an anti-angiogenic activity[15, 16].

*The route of administration*

The bioavailability of a drug is strongly influenced by its route of administration. The injection route of administration (e.g. intravenous) facilitates the drug reaching the bloodstream in a shorter time period. Drug absorption is thereby avoided in other areas and the bioavailability of the drug is increased. Consequently, patients treated with MRONJ-associated drugs, who are administered by injection, would be at a greater risk of developing an adverse event than patients treated with drugs taken *per os*[11, 17, 18].

*Cumulative dose*

A cumulative dose is strongly related to the half-life of a given drug and the duration of treatment (in addition to the affinity of the molecule for the target tissue). BPs persist at the skeletal level for a long period of time, being characterised by a half-life of approximately 10 years[6]. In contrast, denosumab has a short half-life of approximately 32 days, and its effects on bone resorption gradually diminish within six months after its suspension[8]. AA drugs also have a short half-life (e.g. 20 days for bevacizumab, 40-60 hours for sunitinib) and, compared to drugs with antiresorptive activity, they would seem to be characterised by a lower accumulative effect in the bone[11].

*Duration of treatment*

In most cases of MRONJ associated with intravenously-administered BPs, the time range of onset of the adverse event is *within 2 years*. This range tends to increase in patients receiving oral BP therapy (typically after 3 years with a mean of 4.6 years)[6]. The data relating to denosumab is not unanimous: it would appear that patients taking denosumab have a shorter time range of adverse event onset than BPs[8, 17, 19]. This risk would increase further, resulting in a faster time to MRONJ onset, where treatment is switched from BP to denosumab[20].

It has been demonstrated that the simultaneous or subsequent use of AA drugs in patients already exposed to BP would reduce the time of onset of an adverse event, comparing them to patients taking only one type of antiresorptive drug[16]. This time would reduce still further with patients whose treatment involves only drugs with exclusive anti-angiogenic activity, whether it be by injection (approximately 6-7 months) or orally (approximately 16-17 months)[12].

Drug-related risk factors also include concomitant therapies, which can potentially contribute to the MRONJ outbreak. This category includes drugs where it is believed that there is a potential link between intake and increased susceptibility to the risk of developing a disease, namely: chemotherapy and cancer hormone therapy[21–25]; steroid drugs and other drugs inducing secondary osteoporosis (e.g. corticosteroids)[26, 27]; proton pump inhibitors (e.g. omeprazole)[28]; and thalidomide[29].

**Systemic risk factors**

Systemic risk factors include:

- the underlying pathologies closely associated with MRONJ, by virtue of their treatment with the drugs considered to be at risk; and
- other potentially predisposing comorbidities.

***Underlying pathologies closely associated with MRONJ***

The most susceptible patients are those affected by oncological pathologies; greater susceptibility to MRONJ is related to the strictly pharmacological variables detailed above and to the precariousness of the health conditions of these patients. Cancer patients are very often also affected by other pathologies and/or subject to other adjuvant medical treatment, both of which can compromise systemic health[30]. Specifically, the susceptibility to the cancer treatment-induced bone loss (CTIBL) of cancer patients undergoing adjuvant hormone therapy is of note. The prevention of CTIBL with AR drug treatment is initiated at an early stage of cancer treatment, regardless of the appearance of bone metastases, according to recent guidelines[31]. Among the patients undergoing treatment with AA drugs, those with renal carcinoma undergoing treatment with tyrosine kinase inhibitors (either monotherapy or combined treatment with BPs) seem to be particularly at risk of MRONJ[11, 30].

Patients with osteometabolic disease, who are being treated with AR drugs, are at less risk of developing MRONJ than cancer patients due to the pharmacokinetic characteristics of mainly oral therapy (BPs), and, therefore, the reduced cumulative dose to which they are exposed, when compared to cancer patients. However, following the increasing age of many populations of patients in Europe and, consequently, the increased prevalence of forms of osteoporosis (including secondary forms), there has been an increase in the prevalence of osteoporotic disease in the general population[32], which is associated with an increased tendency to prescribe AR drugs. And this is the case even in the preventive phases of the disease, also including treatment by injection, very often without an adequate assessment of the risk/benefit ratio[33].

***Other comorbidities***

All systemic conditions, whether congenital or acquired, which directly and/or indirectly alter bone metabolism and/or predispose it to osteopenia/osteoporosis, are to be considered possible co-factors for the onset of MRONJ. The conditions regarding the risk of MRONJ with reliable data in the literature are: *diabetes mellitus*[34–37]; rheumatoid arthritis[38]; hypercalcaemia/hyperparathyroidism and osteomalacia/hypovitaminosis D[39–41]; chronic renal failure[42]; and other comorbidities (e.g. immunodepression, hypertension, anaemia)[43, 44]. Finally, the anamnestic detection of lifestyle habits (of maximum impact: smoking), once considered as potential risk factors for the onset of MRONJ[45–47], will play only an indirect role, as risk factors in periodontal and/or peri-implant inflammatory pathology. Indeed, it is only the latter which can be said to be the main, direct and local risk factor for MRONJ.

**Local risk factors**

All physiological and/or pathological conditions, which directly or indirectly compromise optimal oral health, especially at the dento-periodontal level (thereby rendering the jaw bone more susceptible to infection and/or mechanical stress) are to be considered as the most important risk factors for the onset of MRONJ. And this is particularly relevant regarding immunodeficient patients and those undergoing long-term treatment by injection.

*Dento-periodontal and peri-implant inflammatory pathology*

The presence of dento-periodontal and/or peri-implant inflammatory pathologies (e.g. periodontitis, odontogenic infections, peri-implantitis), especially where chronic, is a significant risk factor for MRONJ[48–51]. Inflammation is a non-specific and innate defence mechanism, which, although protective, has deleterious effects on the tissues when it becomes chronic, becoming a disease in itself. Affecting bone tissue, inflammation is predisposed to necrosis due to the release of chemical mediators (e.g. cytokines, prostaglandins) and an ischaemic action, the latter caused by oedema and increased intramedullary pressure[52]. The presence of infection has a co-adjuvant role: in addition to having a direct toxic action, many bacteria, including Gram-periodontopathic bacteria are able to stimulate the release of inflammatory prostaglandins, which promote bone resorption. Finally, it is important to emphasise that the persistence of a dento-periodontal and/or peri-implant, infective-inflammatory condition frequently leads to the need for dental surgical procedures (e.g. dental extractions).

*Dento-alveolar surgery*

The link between the performance of dento-alveolar surgical procedures and the occurrence of the adverse events of MRONJ events has been amply studied in the literature[53, 54]. Etiopathogenetic hypotheses can be attributed to the traumatic action exerted during surgery on a bone with an altered metabolism due to the activity of the MRONJ-associated drugs; different mechanisms modify and delay the normal healing processes[55]. An alternative or co-existing hypothesis is that of pre-existing infectious problems (i.e. chronic inflammatory dento-periodontal pathology). Indeed, there are numerous studies which have demonstrated early signs and symptoms of MRONJ, and which are clinically associated with chronic infectious-inflammatory, dento-periodontal processes, prior to teeth extraction/s with a poor prognosis[56–59]. For example, by means of histological investigation of the alveolar bone taken from the same extraction site as compromised teeth, Nicolatou-Galitis *et al* detected the presence of MRONJ in 70% of the selected cases[48]. That is, dento-periodontal infectious-inflammatory processes would be more at risk of the onset of MRONJ (rather than extraction procedures), especially where they are chronic. Signs and symptoms related to dento-periodontal infectious-inflammatory processes should lead to a differential diagnosis of MRONJ (especially with unexposed MRONJ).

*Implant surgery/osseointegrated implantology*. Two different types of adverse events related to implant-prosthetic therapy have been outlined in the literature: MRONJ which is associated with implant surgery (Implant Surgery-Triggered Osteonecrosis or ISTO), and MRONJ which is secondary to the presence of the implant, which has been more or less osseointegrated (Implant Presence-Triggered Osteonecrosis - IPTO)[60]. The first type of adverse event (ISTO) is considered to be strictly dependent on surgically placing the fixture in a bone with an altered, drug-induced metabolism, with onset times ranging from post-operative to within 6 months. The second type of adverse event (IPTO) is considered to be dependent on the appearance of infectious-inflammatory processes, which are promoted by the absence of a typical barrier effect at the bone-implant interface (e.g. peri-implantitis) and/or the development of occlusal microcracks. The latter can be due to the functional masticatory load, with appearance times in the medium-to-long term (over 6 months after surgery)[61, 62]. The latter entity seems to be of greater impact in terms of patient volumes, especially in patient populations subject to monitoring times in excess of 2 years[63].

*Incongruous removable prostheses*. Chronic trauma of incongruous prostheses to the mucosa results in decubitus formation, thereby promoting microbial entry into the underlying tissues with the subsequent development of MRONJ[64–66].

*Predisposing anatomical factors*. The presence of anatomical irregularities (such as tori, exostosis, a particularly pronounced mylohyoid ridge), which are characterised by a physiological thinning of the overlying mucosa, may represent a risk factor for the development of MRONJ, especially in patients with removable total prostheses. Furthermore, it is important to underline that, in most of the case histories reported in the literature, MRONJ is more frequently reported in the mandibular bone than in the maxillary bone. This is probably due to the physiological characteristics of the mandible, which make it more susceptible to complications of an infectious-inflammatory nature and/or mechanical stress (e.g. terminal vascularisation)[18].

Whilst small in number and of dubious reliability, there are reports of MRONJ which have been defined as “spontaneous”, i.e. occurring in patients who have been exposed to MRONJ-related drugs but in the absence of other systemic and local factors. For these forms of MRONJ, genetic factors could play a major role[67]. For a more extensive discussion of risk factors, refer to the recent *Italian Recommendations* for the management of MRONJ[68, 69].

**A classification of patients at risk of MRONJ (risk population clustering)**

On the basis of the systemic pathology for which an MRONJ-associated drug is taken, the following risk groups (in decreasing order) have been identified:

**1a) ONCOLOGICAL AND HAEMATOLOGICAL PATIENTS TREATED WITH ANTIRESORPTIVE DRUGS (BP and/or DENOSUMAB)**

Patients being treated for bone metastases or multiple myeloma, for the prevention of skeletal-related events, undergoing:

- monthly injections of BP and/or denosumab (120 mg), for extensive time periods (i.e. >18-24 months); concomitant and/or subsequent intake of drugs with anti-angiogenic activity; presence of local and/or systemic risk factors;
- monthly injections of BP and/or denosumab (120 mg) of short duration (i.e. <12 months) or quarterly injections of zoledronate; absence of local and systemic risk factors.

MRONJ has been reported among patients treated with antiresorptive drugs, with bone metastases or myeloma, with percentages varying widely from between 1% and 9% (with a probable underestimation, due to restrictive definitions, even in randomised trials)[17]. Generally, the risk is greater for: intravenous administration (vs intramuscular vs oral vs subcutaneous), for prolonged treatment times (≥ cumulative dose), for the presence of systemic and/or drug-related and/or local factors increasing the risk of MRONJ.

**1b)** **ONCOLOGICAL PATIENTS ON TREATMENT WITH DRUGS WITH A PREVALENT ANTI-ANGIOGENETIC ACTIVITY**

Patients may take medication with a prevalent anti-angiogenetic activity, together with anti-resorptive drugs. They will be simultaneously exposed to a greater number of systemic and pharmacological risk factors, which define an elevated risk of developing MRONJ from the initial intake of the MRONJ-related drug[17, 18, 68].

For the purposes of oral prevention protocols, cancer patients in groups 1a) and 1b) are placed in the ONC category and classified, on the basis of their different risk (R), into 3 subgroups with the following abbreviations (Fig.1):

- ONC-R_0_ (if MRONJ-related drug administration is planned but not yet commenced)

- ONC-R_+_ (if MRONJ-related drug administration has been commenced)

- ONC-R_++_ (if taking concomitant or subsequent drugs with anti-angiogenic activity and/or in presence of local and/or systemic risk factors).

**2. ONCOLOGICAL PATIENTS ON HORMONAL THERAPY, BEING TREATED WITH BP and/or DENOSUMAB**

Oncological patients receiving antihormonal therapy for the prevention of fractures related to iatrogenic bone loss (CTIBL). These patients have a lower risk of MRONJ than category 1a) and 1b). These patients are usually women with pre- and post-menopausal breast cancer receiving adjuvant hormone therapy or men with prostate cancer receiving an androgen blockade. The treatment plan with MRONJ-related drugs is comparable to patients with an osteometabolic pathology, and thus also the risk of MRONJ.

**3. PATIENTS WITH A OSTEOMETABOLIC PATHOLOGY, BEING TREATED WITH ANTIRIASSORBIOUS DRUGS (BP and/or DENOSUMAB)**

The estimated prevalence of MRONJ in osteometabolic or osteometabolic-assimilated patients has been generally estimated to range from between 0.02% and 1%[70–74]. However, frequency data for osteometabolic patients is not often directly available as most case series are merged with oncological patients: e.g. patients receiving BP *per os*, compared to the total number of MRONJ cases, accounted for 7.1% and 8.9% respectively in two multicentre studies[75, 76].

Patients with an osteometabolic disease at risk of MRONJ can be distinguished, according to pharmacological and systemic risks, into two subgroups with the following abbreviations (Fig.2)[77]:

- OST-R_0_ (patients not at risk)
- OST-R_x_ (subjects with potentially increased risk, compared to OST-R_0_, although this cannot be defined (x), based on: AR drug type, dosage, treatment duration, possible bone accumulation, presence of oral triggers and coexisting systemic risk factors).

The OST-R_0_ subgroup consists of:

- patients who are or who are not currently being treated with AR drugs (not currently taking this medication)
- patients who have already been treated with AR drugs for less than 3 years without coexisting systemic risk factors

The OST-R_x_ subgroup consists of:

- patients who have been taking AR medication for more than 3 years
- patients taking AR medication for less than 3 years with additional risk factors (i.e. concomitant corticosteroid intake, diabetes, oral triggers).

Furthermore, an OST patient may be dynamically classified in a different risk subgroup from the initial subgroup. Furthermore, it can be hypothesised that the progression of risk in osteometabolic patients does not have a linear progression; the analysis of this risk profile is closely associated with the patient and the coexistence of pharmacological, genetic, systemic and/or local risk factors, which determine the patient’s probability of developing MRONJ[68].

*The Board agrees that all the information described above, regarding primary and secondary prevention (early diagnosis of suspected MRONJ) should be acquired, recorded and updated by the dental hygienist in order to: 1) assist defining the risk profile of patients; and 2) ensure appropriate preventive management.*

**Secondary prevention: the role of the dental hygienist in the diagnosis of suspected MRONJ and timing of the follow-up**

The aim of MRONJ secondary prevention is early diagnosis (i.e. the recognition of all those clinical/radiological signs and/or symptoms), which can be associated with an early stage MRONJ[16, 77].

Patients may be considered to have MRONJ if all the following characteristics are present[16, 68, 69, 78, 79]:

- Current or previous treatment with antiresorptive (AR) or antiangiogenic agents (AA)
- Clinical-radiological findings of progressive bone destruction
- No history of radiation therapy to the jaws
- No presence of cancer lesions (e.g. oral squamous cell carcinoma) or metastatic disease to the jaws.

Of importance, it was recently suggested that patients taking AR medications and presenting with signs of bone necrosis in previously radiated jaws should be regarded as true MRONJ cases if they received less than 40 GY radiation dose at the necrosis site[80]. This suggested radiation dose cut-off  is not included, at present, in any published Expert Panel Recommendation and will require further confirmation.

**Diagnostic work-up of MRONJ**

A diagnostic work-up permits the clinician to make a tentative diagnosis (Step 1) and, by means of a differential diagnosis (Step 2), to reduce the time for final diagnosis by the physician (Step 3) (Fig.3).

***Step 1.*** The dental hygienist and the dentist are the most important figures in the early diagnosis of MRONJ because they both deal with secondary prevention and identifying local risk factors for MRONJ (see ‘Local risk factors’). A suspicion of MRONJ should be raised whenever a patient, who is being treated (currently or previously) with drugs defined as *at risk*, presents signs and symptoms in the oral cavity compatible with MRONJ. The presence of such signs should lead the practitioner to request radiological investigations to confirm or exclude the possibility of disease.

The role of each of the actors involved in the diagnostic process can be summarised as follows[81]:

***Step 2***. A differential diagnosis must consider all those pathological conditions of the oral cavity presenting clinical and radiological signs and/or symptoms overlapping with the initial phases of MRONJ, or that may constitute a precipitating factor for MRONJ. At this stage the dental hygienist plays an important role regarding: the differential diagnosis between periodontal disease, endo-periotic abscesses and early stages of MRONJ, and in referring the patient to the dentist or a specialised centre for the diagnosis of MRONJ for further evaluation and/or to other specialised centres.

***Step 3***. Patients should be referred to specialised centres for the treatment of MRONJ (e.g. outpatient clinics of Oral Medicine and Pathology, Oral Surgery, Maxillofacial Surgery) where more specific radiological and instrumental investigations and a final reassessment (step analysis) can be performed.

**DIAGNOSTIC CRITERIA OF MRONJ**

The need to define a diagnosis of MRONJ on the basis of clinical and radiological criteria (the responsibility of the dentist) has been restated in the most recent clinical recommendations regarding MRONJ. This requirement derives from the fact that a substantial number of patients treated with drugs, which are associated with the risk of MRONJ, present symptoms early and late without bone exposure[82, 83]. While recognising that exposure of the necrotic bone is still the most reliable indicator of MRONJ[17, 53, 84], clinical and instrumental investigations into other non-specific symptoms can and should prompt a suspicion of MRONJ. The preventive action of the dental hygienist is critical at this stage, even where the bone is not exposed, particularly where there is a recognised dento-periodontal lesion[85–88].

***Clinical criteria***

In addition to bone exposure (which is recognised as the most typical clinical symptom of MRONJ), the dental hygienist should be aware that there is a variety of other clinical signs and/or symptoms. Their presence alone or in association with other symptoms should raise the suspicion of the disease of MRONJ and suggest an appropriate and differential diagnosis (Table 2). Symptoms which are usually associated with MRONJ are pain and paraesthesia/dysesthesia of the lips (e.g. Vincent’s symptom)[89]. However, pain does not appear to be present at the onset of MRONJ in a large number of patients[90, 91]. The basis of pain may be different (e.g. neuropathic, infectious, inflammatory, myogenic pain), and it can occur in a variety of ways: odontalgia; gravitational bone pain; myogenic pain; sinus pain; and trigeminal pain (hyperesthesia)[89, 92, 93].

***Radiological criteria***

The dental hygienist should be aware that radiological examination is essential for diagnostic confirmation where MRONJ is suspected; it should be a significant step in diagnostic work-up. However, since no specific radiological signs of MRONJ have been recognised (except bone sequestrum) (Table 3), the diagnosis and staging of the disease must always be associated with corresponding clinical findings. Where MRONJ is suspect, the dental hygienist will refer the patient to the dentist in this diagnostic phase. To date, the most commonly used radiological techniques for the diagnosis of MRONJ can be classified as *Level I investigations* (e.g. orthopantomography)[94, 95] and *Level II investigations* (e.g. computerised tomography)[96, 97].

Their widespread use depends on a number of factors:

- an improved definition of skeletal structures, compared to other known methods
- easier access to radiographical investigations, due to the wide availability of relevant technology
- limited costs
- an effective knowledge of the techniques deployed by the doctor and dentist and
- comparability between specialists.

Together with intraoral radiography, orthopantomography (OPT) is a tool for the obtaining with ease of general information relating to the mandible and maxillary bone, particularly in identifying any bone sequestration and osteolytic areas, combined with areas of osteosclerosis. By means of these investigative tools, it is possible to highlight various subclinical signs of osteonecrosis (e.g. enlargement of the periodontal space) or an alteration/heterogeneity of the trabecular bone, which may be related to early development of osteomyelitis/osteonecrosis process. However, these tools do not permit the differentiation of osteolytic lesions from possible bone metastases, especially if they are isolated. Nevertheless, the use of OPT as an initial tool for investigating the patient with suspected ONJ is also useful in ascertaining the state of dental-periodontal health. The dental hygienist can, therefore, facilitate the diagnosis of suspected MRONJ at the initial assessment of periodontal conditions deploying OPT.

*Computerised tomography (CT)*, particularly spiral CT, and *Cone Beam Computerised Tomography (CBCT*) have a higher contrast resolution than conventional radiography; both types of CT provide detailed information regarding the number and nature of any osteolytic and osteosclerotic lesions present[96, 98]. They are indispensable for investigating the cortical and trabecular bones. CT may discriminate to an acceptable degree of approximation between healthy and pathological bone tissue in many cases, providing precise information as to the extent of the disease[97]. CBCT has recently been accredited as an instrumental method facilitating earlier detection of the initial stages of MRONJ disease than would be the case with DPR. It should be noted that not all CBCT equipment is suitable: only equipment with FOV widths, including upper and lower jaws in the field of view, can provide diagnostic information relating to bone lesions, which is comparable to traditional CT[99].

*In light of the above, the Board believes that OPT and CT (conventional or CBCT) are to be considered as the most useful First and Second Level investigation techniques respectively, in defining a routine diagnostic pathway where MRONJ is suspect.*

The most significant radiological patterns (for intraoral X-ray/OPT and CT/CBCT), which are compatible with a diagnosis of MRONJ, are reported in (Table 3). They have been divided into early and late stages[98, 100]. This distinction, maintained in this updated document, warrants further clarification, as below:

- the absence of radiological signs is characteristic to MRONJ; any signs must always be correlated with one or more clinical criteria in order to investigate the possibility of MRONJ in reaching a conclusive diagnosis;
- the persistence of a post-extraction alveolus and the thickening of the *lamina dura* after dental extraction may be considered an early sign of MRONJ by OPT. However, there are some studies which have reported the persistence of the post-extraction alveolus one year after tooth extraction in patients who did not show any clinical signs of MRONJ[92]. Indeed, according to Saia *et al*., the absence of alveolus repair is linked to the inhibitory action of bone remodelling at the extraction site, and when the absence of alveolus repair may last for several years, even without clinical signs of disease[92]. Therefore, the persistence of one or more post-extraction alveoli at OPT cannot always be considered a sign of disease in the short-term period following tooth avulsions.
- CT can be considered the most effective way by which to identify early changes in bone marrow structure, which are compatible with MRONJ[92, 96, 98, 101, 102]. Late signs of MRONJ, particularly bone sequestrum, diffuse osteosclerosis, periosteal reaction and pathological fractures are all equally recognizable by OPT and CT scans; the latter (CT scan) displays increased sensitivity in defining the extent of the osteonecrotic process, especially regarding the identification of any fistulous pathways[102, 103].

In addition to OPT and CT/CBCT, there exist further radiological investigations, which are available for the evaluation of MRONJ. These Level II examinations include several radiological investigations: Magnetic Resonance Imaging (MRI); total-body bone scintigraphy with CT[104, 105]; scintigraphy with 99mTc-labelled leukocytes; positron emission tomography or single-photon emission tomography. For a more extensive discussion of these radiological examinations, refer to the recent Italian Recommendations regarding the management of MRONJ[68].

*The Board concurs that the recording and reporting of clinical observations by the dental hygienist, which could possibly be attributable to MRONJ and the need to conduct or propose appropriate radiological investigations, should always be considered in patient management. This is to ensure diagnostic confirmation of MRONJ in avoiding underestimating the possibility of a diagnosis of MRONJ and to encourage the implementation of preventive measures. The intrinsic characteristics of the dental hygienist profile, attuned to the diagnosis of suspected oral pathologies, will be able to contribute to reducing the time necessary for making a definitive diagnosis of MRONJ. It is the considered opinion of the authors of this research that an understanding of the pathology and its risk factors will enrich the skills of the dental hygienist working in the field of oral prevention, thereby enhancing preventive care for patients undergoing drug treatment at risk of MRONJ.*

**CLINICAL-RADIOLOGICAL STAGING OF MRONJ**

Although the American classification system proposed by the AAOMS is currently the most widespread and used system in the international scientific community, several doubts regarding a number of their Position Papers have been expressed. This is due to possibility of the AAOMS diagnostic criteria underestimating the incidence of MRONJ due to taxonomic and classification criteria. The latter are considered by many to be too restrictive and exclusive for a disease with extremely heterogeneous and non-specific features such as MRONJ[18, 106].

*The Board, therefore, proposes a classification scheme of MRONJ, in which patients are divided into three stages on the basis of specific clinical and radiological criteria (Table 4).*

The majority of early forms of osteonecrosis in the focal disease stage (Stage 1) are typically characterised by selective bone involvement of the dento-alveolar process.

Stage 1 regards early forms of osteonecrosis, which are typically characterised by selective bone involvement of the dento-alveolar process.

Stage 2 includes all diffuse forms of MRONJ, and Stage 3 includes all complex forms of MRONJ. Stages 1 and 2 are further subdivided into (a) asymptomatic and (b) symptomatic forms. These are based on the presence or absence of pain and/or suppuration, which are considered independent variables of the extent of the osteonecrosis[107].

*The Board agrees that all the information described above must be acquired and recorded by the dental hygienist, in the primary and secondary prevention phases (early diagnosis of suspected MRONJ) in order to: 1) assist defining the risk profile of patients; and 2) provide appropriate preventive management.*

**References**

1. Buser, D., Dahlin, C. and Schenk R (1994) Guided bone regeneration in implant dentistry. Quintessence Publishing Co., Chicago, pp 49–100

2. Marx RE (2003) Pamidronate (Aredia) and zoledronate (Zometa) induced avascular necrosis of the jaws: A growing epidemic. J Oral Maxillofac Surg 61:1115–1117. https://doi.org/10.1016/S0278-2391(03)00720-1

3. Bagan J V., Murillo J, Jimenez Y, et al (2005) Avascular jaw osteonecrosis in association with cancer chemotherapy: Series of 10 cases. J Oral Pathol Med 34:120–123. https://doi.org/10.1111/j.1600-0714.2004.00269.x

4. Eckert AW, Maurer P, Meyer L, et al (2007) Bisphosphonate-related jaw necrosis - Severe complication in Maxillofacial surgery. Cancer Treat. Rev. 33:58–63

5. Marc Baltensperger (2009) Osteomyelitis of the Jaws. In: (Eds) S (ed). pp 5–36

6. Fung P, Bedogni G, Bedogni A, et al (2017) Time to onset of bisphosphonate-related osteonecrosis of the jaws: a multicentre retrospective cohort study. Oral Dis 23:477–483. https://doi.org/10.1111/odi.12632

7. Mücke T, Krestan CR, Mitchell DA, et al (2016) Bisphosphonate and medication-related osteonecrosis of the jaw: A review. Semin. Musculoskelet. Radiol. 20:305–314

8. de Oliveira CC, Brizeno LAC, de Sousa FB, et al (2016) Osteonecrosis of the jaw induced by receptor activator of nuclear factor-kappa B ligand (Denosumab) - Review. Med. Oral Patol. Oral Cir. Bucal 21:e431–e439

9. Troeltzsch M, Woodlock T, Kriegelstein S, et al (2012) Physiology and pharmacology of nonbisphosphonate drugs implicated in osteonecrosis of the jaw. J Can Dent Assoc 78:c85

10. Hamadeh IS, Ngwa BA, Gong Y (2015) Drug induced osteonecrosis of the jaw. Cancer Treat. Rev. 41:455–464

11. Fusco V, Santini D, Armento G, et al (2016) Osteonecrosis of jaw beyond antiresorptive (bone-targeted) agents: new horizons in oncology. Expert Opin Drug Saf 15:925–935. https://doi.org/10.1080/14740338.2016.1177021

12. Pimolbutr K, Porter S, Fedele S (2018) Osteonecrosis of the Jaw Associated with Antiangiogenics in Antiresorptive-Naïve Patient: A Comprehensive Review of the Literature. Biomed Res Int 2018:1–14. https://doi.org/10.1155/2018/8071579

13. Nicolatou-Galitis O, Kouri M, Papadopoulou E, et al (2019) Osteonecrosis of the jaw related to non-antiresorptive medications: a systematic review. Support Care Cancer 27:383–394. https://doi.org/10.1007/s00520-018-4501-x

14. Saran U, Gemini Piperni S, Chatterjee S (2014) Role of angiogenesis in bone repair. Arch Biochem Biophys 561:109–17. https://doi.org/10.1016/j.abb.2014.07.006

15. Zhang X, Hamadeh IS, Song S, et al (2016) Osteonecrosis of the Jaw in the United States Food and Drug Administration’s Adverse Event Reporting System (FAERS). J Bone Min Res 31:336–340. https://doi.org/10.1002/jbmr.2693

16. Campisi G, Fedele S, Fusco V, et al (2014) Epidemiology, clinical manifestations, risk reduction and treatment strategies of jaw osteonecrosis in cancer patients exposed to antiresorptive agents. Futur Oncol 10:257–275. https://doi.org/10.2217/fon.13.211

17. Ruggiero SL, Dodson TB, Fantasia J, et al (2014) American Association of Oral and Maxillofacial Surgeons position paper on medication-related osteonecrosis of the jaw--2014 update. J Oral Maxillofac Surg 72:1938–1956. https://doi.org/10.1016/j.joms.2014.04.031

18. Schiodt M, Otto S, Fedele S, et al (2019) Workshop of European task force on medication-related osteonecrosis of the jaw—Current challenges. Oral Dis 25:1815–1821. https://doi.org/10.1111/odi.13160

19. Watts NB, Grbic JT, Binkley N, et al (2019) Invasive oral procedures and events in postmenopausal women with osteoporosis treated with denosumab for up to 10 years. J Clin Endocrinol Metab 104:2443–2452. https://doi.org/10.1210/jc.2018-01965

20. Yarom N, Lazarovici TS, Whitefield S, et al (2018) Rapid onset of osteonecrosis of the jaw in patients switching from bisphosphonates to denosumab. Oral Surg Oral Med Oral Pathol Oral Radiol 125:27–30. https://doi.org/10.1016/j.oooo.2017.09.014

21. Ponnapakkam T, Katikaneni R, Nichols T, et al (2011) Prevention of chemotherapy-induced osteoporosis by cyclophosphamide with a long-acting form of parathyroid hormone. J Endocrinol Invest 34:e392-7. https://doi.org/10.3275/7864

22. Vanderschueren D, Laurent MR, Claessens F, et al (2014) Sex steroid actions in male bone

23. Shao YH, Moore DF, Shih W, et al (2013) Fracture after androgen deprivation therapy among men with a high baseline risk of skeletal complications. BJU Int 111:745–752. https://doi.org/10.1111/j.1464-410X.2012.11758.x

24. Edwards BJ, Gradishar WJ, Smith ME, et al (2016) Elevated incidence of fractures in women with invasive breast cancer. Osteoporos Int 27:499–507. https://doi.org/10.1007/s00198-015-3246-3

25. Pedersini R, Monteverdi S, Mazziotti G, et al (2017) Morphometric vertebral fractures in breast cancer patients treated with adjuvant aromatase inhibitor therapy: A cross-sectional study. Bone 97:147–152. https://doi.org/10.1016/j.bone.2017.01.013

26. Iwamoto J, Takeda T, Sato Y (2005) Prevention and treatment of corticosteroid-induced osteoporosis. Yonsei Med J 46:456–63. https://doi.org/10.3349/ymj.2005.46.4.456

27. Frenkel B, White W, Tuckermann J (2015) Glucocorticoid-Induced osteoporosis. 872:179–215. https://doi.org/10.1007/978-1-4939-2895-8_8

28. Thong BKS, Ima-Nirwana S, Chin KY (2019) Proton pump inhibitors and fracture risk: A review of current evidence and mechanisms involved. Int. J. Environ. Res. Public Health 16

29. Song Z, Dong W, Yin L, et al (2015) Effect of thalidomide on development of bisphosphonate-related osteonecrosis of the jaws in rats. Nan Fang Yi Ke Da Xue Xue Bao 35:1084–1089. https://doi.org/10.3969/j.issn.1673-4254.2015.08.02

30. Schiodt M, Vadhan-Raj S, Chambers MS, et al (2018) A multicenter case registry study on medication-related osteonecrosis of the jaw in patients with advanced cancer. Support Care Cancer 26:1905–1915. https://doi.org/10.1007/s00520-017-4003-2

31. Medica O, Grottaferrata CINI, Greco C (2019) Linee guida AIOM - Trattamento delle metastasi ossee

32. Soares AL, Simon S, Gebrim LH, et al (2020) Prevalence and risk factors of medication-related osteonecrosis of the jaw in osteoporotic and breast cancer patients: a cross-sectional study. Support Care Cancer 28:2265–2271. https://doi.org/10.1007/s00520-019-05044-0

33. SIE, SIGG, SIMFER, SIMG, SIMI, SIOMMMS , SIR S (2018) Linee Guida sulla gestione dell’Osteoporosi e delle Fratture da fragilità

34. Peer A, Khamaisi M (2015) Diabetes as a risk factor for medication-related osteonecrosis of the jaw. J Dent Res 94:252–60. https://doi.org/10.1177/0022034514560768

35. Molcho S, Peer A, Berg T, et al (2013) Diabetes microvascular disease and the risk for bisphosphonate-related osteonecrosis of the jaw: A single center study. J Clin Endocrinol Metab 98:1807–1812. https://doi.org/10.1210/jc.2013-2434

36. Paek SJ, Park W-J, Shin H-S, et al (2016) Diseases having an influence on inhibition of angiogenesis as risk factors of osteonecrosis of the jaw. J Korean Assoc Oral Maxillofac Surg 42:271–277. https://doi.org/10.5125/jkaoms.2016.42.5.271

37. Valenzuela L, Alonso-Bouzõn C, Mañas LR, et al (2015) Bisphosphonate-related osteonecrosis of the jaw in an 80-year-old woman with diabetes mellitus: Case report. Blackwell Publishing Inc.

38. Di Fede O, Bedogni A, Giancola F, et al (2016) BRONJ in patients with rheumatoid arthritis: a multicenter case series. Oral Dis 22:543–548. https://doi.org/10.1111/odi.12490

39. Bedogni A, Saia G, Bettini G, et al (2012) Osteomalacia: the missing link in the pathogenesis of bisphosphonate-related osteonecrosis of the jaws? Oncologist 17:1114–9. https://doi.org/10.1634/theoncologist.2012-0141

40. Leizaola-Cardesa I-O, Aguilar-Salvatierra A, Gonzalez-Jaranay M, et al (2016) Bisphosphonates, vitamin D, parathyroid hormone, and osteonecrosis of the jaw. Could there be a missing link? Med Oral Patol Oral Cir Bucal 21:e236-40. https://doi.org/10.4317/medoral.20927

41. Bedogni A, Bettini G, Bedogni G, et al (2019) Is vitamin D deficiency a risk factor for osteonecrosis of the jaw in patients with cancer? A matched case–control study. J Cranio-Maxillofacial Surg 47:1203–1208. https://doi.org/10.1016/j.jcms.2019.03.007

42. Jadu F, Lee L, Pharoah M, et al (2007) A retrospective study assessing the incidence, risk factors and comorbidities of pamidronate-related necrosis of the jaws in multiple myeloma patients. Ann Oncol 18:2015–2019. https://doi.org/10.1093/annonc/mdm370

43. Woo S-B, Hellstein JW, Kalmar JR (2006) Narrative [corrected] review: bisphosphonates and osteonecrosis of the jaws. Ann Intern Med 144:753–61

44. Saad F, Brown JE, Van Poznak C, et al (2012) Incidence, risk factors, and outcomes of osteonecrosis of the jaw: integrated analysis from three blinded active-controlled phase III trials in cancer patients with bone metastases. Ann Oncol 23:1341–1347. https://doi.org/10.1093/annonc/mdr435

45. Mücke T, Haarmann S, Wolff K-D, Hölzle F (2009) Bisphosphonate related osteonecrosis of the jaws treated by surgical resection and immediate osseous microvascular reconstruction. J Craniomaxillofac Surg 37:291–7. https://doi.org/10.1016/j.jcms.2008.12.004

46. Wessel JH, Dodson TB, Zavras AI (2008) Zoledronate, smoking, and obesity are strong risk factors for osteonecrosis of the jaw: a case-control study. J Oral Maxillofac Surg 66:625–31. https://doi.org/10.1016/j.joms.2007.11.032

47. Katz J, Gong Y, Salmasinia D, et al (2011) Genetic polymorphisms and other risk factors associated with bisphosphonate induced osteonecrosis of the jaw. Int J Oral Maxillofac Surg 40:605–611. https://doi.org/10.1016/j.ijom.2011.02.002

48. Nicolatou‐Galitis O, Papadopoulou E, Vardas E, et al (2020) Alveolar bone histological necrosis observed prior to extractions in patients, who received bone‐targeting agents. Oral Dis 26:955–966. https://doi.org/10.1111/odi.13294

49. Rasmusson L, Abtahi J (2014) Bisphosphonate associated osteonecrosis of the jaw: An update on pathophysiology, risk factors, and treatment. Int J Dent 2014:471035. https://doi.org/10.1155/2014/471035

50. Topaloglu G, Koseoglu OT, Karaca C, Kosemehmetoglu K (2017) The effect of chronic dental inflammation on development of Stage 0 medication-related osteonecrosis of the jaw. J Cranio-Maxillofacial Surg 45:1158–1164. https://doi.org/10.1016/j.jcms.2017.05.003

51. Rao NJ, Wang JY, Yu RQ, et al (2017) Role of Periapical Diseases in Medication-Related Osteonecrosis of the Jaws. Biomed Res Int 2017:. https://doi.org/10.1155/2017/1560175

52. Sedghizadeh PP, Kumar SKS, Gorur A, et al (2008) Identification of microbial biofilms in osteonecrosis of the jaws secondary to bisphosphonate therapy. J Oral Maxillofac Surg 66:767–75. https://doi.org/10.1016/j.joms.2007.11.035

53. Khan AA, Morrison A, Hanley DA, et al (2015) Diagnosis and management of osteonecrosis of the jaw: A systematic review and international consensus. J Bone Miner Res 30:3–23. https://doi.org/10.1002/jbmr.2405

54. Nicolatou-Galitis O, Schiødt M, Mendes RA, et al (2019) Medication-related osteonecrosis of the jaw: definition and best practice for prevention, diagnosis, and treatment. Oral Surg Oral Med Oral Pathol Oral Radiol 127:117–135. https://doi.org/10.1016/j.oooo.2018.09.008

55. Gaudin E, Seidel L, Bacevic M, et al (2015) Occurrence and risk indicators of medication-related osteonecrosis of the jaw after dental extraction: a systematic review and meta-analysis. J Clin Periodontol 42:922–932. https://doi.org/10.1111/jcpe.12455

56. Aghaloo TL, Kang B, Sung EC, et al (2011) Periodontal disease and bisphosphonates induce osteonecrosis of the jaws in the rat. J Bone Miner Res 26:1871–1882. https://doi.org/10.1002/jbmr.379

57. Nicolatou-Galitis O, Razis E, Galiti D, et al (2015) Periodontal disease preceding osteonecrosis of the jaw (ONJ) in cancer patients receiving antiresorptives alone or combined with targeted therapies: Report of 5 cases and literature review. Oral Surg Oral Med Oral Pathol Oral Radiol 120:699–706. https://doi.org/10.1016/j.oooo.2015.08.007

58. Hasegawa T, Kawakita A, Ueda N, et al (2017) A multicenter retrospective study of the risk factors associated with medication-related osteonecrosis of the jaw after tooth extraction in patients receiving oral bisphosphonate therapy: can primary wound closure and a drug holiday really prevent MRONJ? Osteoporos Int 28:2465–2473. https://doi.org/10.1007/s00198-017-4063-7

59. Jeong H-GG, Hwang JJ, Lee J-HH, et al (2017) Risk factors of osteonecrosis of the jaw after tooth extraction in osteoporotic patients on oral bisphosphonates. Imaging Sci Dent 47:45–50. https://doi.org/10.5624/isd.2017.47.1.45

60. Giovannacci I, Meleti M, Manfredi M, et al (2016) Medication-related osteonecrosis of the jaw around dental implants: Implant surgery-triggered or implant presence-triggered osteonecrosis? J Craniofac Surg 27:697–701. https://doi.org/10.1097/SCS.0000000000002564

61. Lazarovici TS, Yahalom R, Taicher S, et al (2010) Bisphosphonate-Related Osteonecrosis of the Jaw Associated With Dental Implants. J Oral Maxillofac Surg 68:790–796. https://doi.org/10.1016/j.joms.2009.09.017

62. Guazzo R, Sbricoli L, Ricci S, et al (2017) Medication-Related Osteonecrosis of the Jaw and Dental Implants Failures: A Systematic Review. J Oral Implantol 43:51–57. https://doi.org/10.1563/aaid-joi-16-00057

63. Escobedo MF, Cobo JL, Junquera S, et al (2020) Medication-related osteonecrosis of the jaw. Implant presence-triggered osteonecrosis: Case series and literature review. J Stomatol Oral Maxillofac Surg 121:40–48. https://doi.org/10.1016/j.jormas.2019.04.012

64. Levin L, Laviv A, Schwartz-Arad D (2007) Denture-related osteonecrosis of the maxilla associated with oral bisphosphonate treatment. J Am Dent Assoc 138:1218–1220. https://doi.org/10.14219/jada.archive.2007.0346

65. Hasegawa Y, Kawabe M, Kimura H, et al (2012) Influence of dentures in the initial occurrence site on the prognosis of bisphosphonate-related osteonecrosis of the jaws: a retrospective study. Oral Surg Oral Med Oral Pathol Oral Radiol 114:318–324. https://doi.org/10.1016/j.oooo.2012.04.002

66. Martini V, Bonacii RM, Varani EM, et al (2018) Osteonecrosi dei mascellari indotta da farmaci e protesi mobili: Casi clinici. Dent Cadmos 86:51–60. https://doi.org/10.19256/d.cadmos.01.2018.06

67. Yang G, Singh S, Chen Y, et al (2019) Pharmacogenomics of osteonecrosis of the jaw. Bone 124:75–82

68. Campisi, G; Bedogni, A; Fusco V (2020) Raccomandazioni clinico-terapeutiche sull’osteonecrosi delle ossa mascellari (ONJ) farmaco-relata e sua prevenzione. Palermo University Press, Palermo

69. Campisi G, Mauceri R, Bertoldo F, et al (2020) Medication-related osteonecrosis of jaws (MRONJ) prevention and diagnosis: Italian consensus update 2020. Int J Environ Res Public Health 17:1–15. https://doi.org/10.3390/ijerph17165998

70. Mavrokokki T, Cheng A, Stein B, Goss A (2007) Nature and frequency of bisphosphonate-associated osteonecrosis of the jaws in Australia. J Oral Maxillofac Surg 65:415–23. https://doi.org/10.1016/j.joms.2006.10.061

71. Khosla S, Burr D, Cauley J, et al (2007) Bisphosphonate-associated osteonecrosis of the jaw: Report of a Task Force of the American Society for Bone and Mineral Research. J Bone Miner Res 22:1479–1491. https://doi.org/10.1359/jbmr.0707onj

72. King AE, Umland EM (2008) Osteonecrosis of the jaw in patients receiving intravenous or oral bisphosphonates. Pharmacotherapy 28:667–77. https://doi.org/10.1592/phco.28.5.667

73. Rizzoli R, Burlet N, Cahall D, et al (2008) Osteonecrosis of the jaw and bisphosphonate treatment for osteoporosis. Bone 42:841–847

74. Lo JC, O’Ryan FS, Gordon NP, et al (2010) Prevalence of Osteonecrosis of the Jaw in Patients With Oral Bisphosphonate Exposure. J Oral Maxillofac Surg 68:243–253. https://doi.org/10.1016/j.joms.2009.03.050

75. Yarom N, Yahalom R, Shoshani Y, et al (2007) Osteonecrosis of the jaw induced by orally administered bisphosphonates: Incidence, clinical features, predisposing factors and treatment outcome. Osteoporos Int 18:1363–1370. https://doi.org/10.1007/s00198-007-0384-2

76. Otto S, Abu-Id MH, Fedele S, et al (2011) Osteoporosis and bisphosphonates-related osteonecrosis of the jaw: not just a sporadic coincidence--a multi-centre study. J Craniomaxillofac Surg 39:272–7. https://doi.org/10.1016/j.jcms.2010.05.009

77. Di Fede O, Panzarella V, Mauceri R, et al (2018) The dental management of patients at risk of medication-related osteonecrosis of the jaw: New paradigm of primary prevention. Biomed Res Int 2018:1–10. https://doi.org/10.1155/2018/2684924

78. Bedogni A, Fusco V, Agrillo A, Campisi G (2012) Learning from experience. Proposal of a refined definition and staging system for bisphosphonate-related osteonecrosis of the jaw (BRONJ). Oral Dis 18:621–623. https://doi.org/10.1111/j.1601-0825.2012.01903.x

79. Mauceri R, Toro C, Panzarella V, et al (2021) Oral Squamous Cell Carcinoma Mimicking Medication-Related Osteonecrosis of the Jaws (MRONJ): A Case Series. Oral 2021, Vol 1, Pages 326-331 1:326–331. https://doi.org/10.3390/ORAL1040032

80. Zadik Y, Ganor Y, Rimon O, et al (2021) Assessment of jaw osteonecrosis diagnostic criteria in cancer patients with a history of radiation therapy and exposure to bone-modifying agents. Radiother Oncol 156:275–280. https://doi.org/10.1016/j.radonc.2020.12.026

81. Lo Russo L, Lo Muzio L, Buccelli C, et al (2013) Bisphosphonates-related osteonecrosis of the jaws: Medicolegal issues. Oral Dis 19:425–426. https://doi.org/10.1111/odi.12049

82. Yarom N, Fedele S, Lazarovici TS, Elad S (2010) Is Exposure of the Jawbone Mandatory for Establishing the Diagnosis of Bisphosphonate-Related Osteonecrosis of the Jaw? J Oral Maxillofac Surg 68:705. https://doi.org/10.1016/j.joms.2009.07.086

83. Migliario M, Mergoni G, Vescovi P, et al (2017) Osteonecrosis of the Jaw (ONJ) in Osteoporosis Patients: Report of Delayed Diagnosis of a Multisite Case and Commentary about Risks Coming from a Restricted ONJ Definition. Dent J 5:13. https://doi.org/10.3390/dj5010013

84. Ruggiero SL, Dodson TB, Assael LA, et al (2009) American Association of Oral and Maxillofacial Surgeons Position Paper on Bisphosphonate-Related Osteonecrosis of the Jaws-2009 Update. J Oral Maxillofac Surg 67:2–12. https://doi.org/10.1016/j.joms.2009.01.009

85. Junquera L, Gallego L (2008) Nonexposed Bisphosphonate-Related Osteonecrosis of the Jaws: Another Clinical Variant? J Oral Maxillofac Surg 66:1516–1517. https://doi.org/10.1016/j.joms.2008.02.012

86. Mawardi H, Giro G, Kajiya M, et al (2011) A Role of Oral Bacteria in Bisphosphonate-induced Osteonecrosis of the Jaw. J Dent Res 90:1339–1345. https://doi.org/10.1177/0022034511420430

87. Patel S, Choyee S, Uyanne J, et al (2012) Non-exposed bisphosphonate-related osteonecrosis of the jaw: a critical assessment of current definition, staging, and treatment guidelines. Oral Dis 18:625–32. https://doi.org/10.1111/j.1601-0825.2012.01911.x

88. Koth VS, Figueiredo MA, Salum FG, Cherubini K (2016) Bisphosphonate-related osteonecrosis of the jaw: From the sine qua non condition of bone exposure to a non-exposed BRONJ entity. Dentomaxillofacial Radiol. 45

89. Miksad RA, Lai K-C, Dodson TB, et al (2011) Quality of Life Implications of Bisphosphonate-Associated Osteonecrosis of the Jaw. Oncologist 16:121–132. https://doi.org/10.1634/theoncologist.2010-0183

90. Ruggiero SL, Mehrotra B, Rosenberg TJ, Engroff SL (2004) Osteonecrosis of the jaws associated with the use of bisphosphonates: a review of 63 cases. J Oral Maxillofac Surg 62:527–534. https://doi.org/10.1016/j.joms.2004.02.004

91. Ruggiero SL, Fantasia J, Carlson E (2006) Bisphosphonate-related osteonecrosis of the jaw: background and guidelines for diagnosis, staging and management. Oral Surg Oral Med Oral Pathol Oral Radiol Endod 102:433–41. https://doi.org/10.1016/j.tripleo.2006.06.004

92. Saia G, Blandamura S, Bettini G, et al (2010) Occurrence of bisphosphonate-related osteonecrosis of the jaw after surgical tooth extraction. J Oral Maxillofac Surg 68:797–804. https://doi.org/10.1016/j.joms.2009.10.026

93. Bedogni A, Saia G, Bettini G, et al (2011) Long-term outcomes of surgical resection of the jaws in cancer patients with bisphosphonate-related osteonecrosis. Oral Oncol 47:420–424. https://doi.org/10.1016/j.oraloncology.2011.02.024

94. Sanna G, Preda L, Bruschini R, et al (2006) Bisphosphonates and jaw osteonecrosis in patients with advanced breast cancer. Ann Oncol Off J Eur Soc Med Oncol 17:1512–6. https://doi.org/10.1093/annonc/mdl163

95. Mawardi H, Treister N, Richardson P, et al (2009) Sinus tracts--an early sign of bisphosphonate-associated osteonecrosis of the jaws? J Oral Maxillofac Surg 67:593–601. https://doi.org/10.1016/j.joms.2008.09.031

96. Chiandussi S, Biasotto M, Dore F, et al (2006) Clinical and diagnostic imaging of bisphosphonate-associated osteonecrosis of the jaws. Dentomaxillofac Radiol 35:236–43. https://doi.org/10.1259/dmfr/27458726

97. Mallya SM, Tetradis S (2018) Imaging of Radiation- and Medication-Related Osteonecrosis. Radiol Clin North Am 56:77–89. https://doi.org/10.1016/j.rcl.2017.08.006

98. Bianchi SD, Scoletta M, Cassione FB, et al (2007) Computerized tomographic findings in bisphosphonate-associated osteonecrosis of the jaw in patients with cancer. Oral Surgery, Oral Med Oral Pathol Oral Radiol Endodontology 104:249–258. https://doi.org/10.1016/j.tripleo.2007.01.040

99. Leite AF, Ogata FDS, Melo NS De, Figueiredo PTDS (2014) Imaging findings of bisphosphonate-related osteonecrosis of the jaws: A critical review of the quantitative studies. Int J Dent 2014:784348. https://doi.org/10.1155/2014/784348

100. Krishnakumar N, Sulfikkarali NK, Manoharan S, Venkatachalam P (2013) Raman spectroscopic investigation of the chemopreventive response of naringenin and its nanoparticles in DMBA-induced oral carcinogenesis. Spectrochim Acta - Part A Mol Biomol Spectrosc 115:648–653. https://doi.org/10.1016/j.saa.2013.05.076

101. Bedogni A, Blandamura S, Lokmic Z, et al (2008) Bisphosphonate-associated jawbone osteonecrosis: a correlation between imaging techniques and histopathology. Oral Surg Oral Med Oral Pathol Oral Radiol Endod 105:358–364. https://doi.org/10.1016/j.tripleo.2007.08.040

102. Morag Y, Morag-Hezroni M, Jamadar DA, et al (2009) Bisphosphonate-related osteonecrosis of the jaw: a pictorial review. Radiographics 29:1971–84. https://doi.org/10.1148/rg.297095050

103. Phal PM, Myall RWT, Assael LA, Weissman JL (2007) Imaging findings of bisphosphonate-associated osteonecrosis of the jaws. AJNR Am J Neuroradiol 28:1139–1145. https://doi.org/10.3174/ajnr.A0518

104. Bertoldo F, Santini D, Lo Cascio V (2007) Bisphosphonates and osteomyelitis of the jaw: a pathogenic puzzle. Nat Clin Pract Oncol 4:711–21. https://doi.org/10.1038/ncponc1000

105. O’Ryan FS, Khoury S, Liao W, et al (2009) Intravenous bisphosphonate-related osteonecrosis of the jaw: bone scintigraphy as an early indicator. J Oral Maxillofac Surg 67:1363–72. https://doi.org/10.1016/j.joms.2009.03.005

106. Fedele S, Bedogni G, Scoletta M, et al (2015) Up to a quarter of patients with osteonecrosis of the jaw associated with antiresorptive agents remain undiagnosed. Br J Oral Maxillofac Surg 53:13–17. https://doi.org/10.1016/j.bjoms.2014.09.001

107. Tronchet, A., Bettini, G. et al (2011) Studio dell’estensione clinico-radiologica dell’osteonecrosi dei mascellari associata a bisfosfonati. Atti del XVII Congr Naz della Soc Ital di Chir Maxillo-Facciale (SICMF), Como

**Table 1.** Risk factors for MRONJ (SICMF-SIPMO)[68]

| Drug-related | Molecule type | 1. predominantly antiresorptive activity (AR) 2. predominantly anti-angiogenic activity (AA) |
| --- | --- | --- |
|  | Route of administration | 1. endovenous 2. intramuscular 3. subcutaneous 4. oral |
|  | Cumulative dose |  |
|  | Duration of treatment |  |
|  | Concomitant therapies | 1. chemotherapy and anticancer hormone therapy 2. steroid drugs and other drugs inducing secondary osteoporosis 3. thalidomide |
| System | Underlying pathology | 1. oncology 2. osteometabolic |
|  | Comorbidities | 1. *Diabetes mellitus* 2. Rheumatoid arthritis 3. Hypercalcaemia/ hyperparathyroidism and osteomalacia/ hypovitaminosis D 4. Chronic kidney failure 5. Lifestyle habits (e.g. smoking) 6. Other comorbidities |
| Local | Chronic inflammatory dento-periodontal and peri-implant pathology  Dento-alveolar surgery  Implant surgery/osseointegrated implantology  Incongruous removable prostheses  Predisposing anatomical factors: a) palatal tori  b) exostosis  c) pronounced mylohyoid ridge | |
|  |  |  |

**Table 2.** Non-specific clinical criteria for the diagnosis of MRONJ (SICMF-SIPMO)[68]

| **Clinical signs and symptoms** | |
| --- | --- |
| - halitosis - dental abscess - mandibular asymmetry - pain: dental and/or bone origin - exposure of necrotic bone - mucosal fistula - extra-oral fistula - mucosal hyperemia - failure of post-extraction, alveolar mucosal repair | - rapid onset of dental mobility - preternatural mandibular mobility (with/out malocclusion) - paresthesia/dysesthesia of the lips* - fluid discharge from the nose - purulent discharge - spontaneous abduction of bone fragments - trismus - soft tissue swelling |

* due to irritation of the inferior alveolar nerve/infraorbital nerve

**Table 3.** Radiological criteria for the diagnosis of MRONJ - OPT and CT scans (SICMF-SIPMO)[68]

| **Non-specific radiological symptoms of MRONJ** | | |
| --- | --- | --- |
|  | **early stages** | **late stages** |
| **Level I** | - thickening of the alveolar ridge and   *lamina dura* sclerosis   - persistence of post-extraction alveolus - *sequestra* - periodontal space widening | - pathological fracture - thickening of the inferior alveolar nerve canal - diffuse osteosclerosis - maxillary sinus opacification - periosteal reaction |
| **Level II** | - cortical erosion - thickening of the alveolar ridge and *lamina dura* sclerosis - trabecular thickening - focal medullary osteosclerosis*** - persistence of post-extraction alveolar - *sequestra* - periodontal space widening | - oroantral, oronasal, muco-cutaneous fistulae - pathological fracture - thickening of the inferior alveolar nerve canal - osteolysis extending into the maxillary sinus - diffuse osteosclerosis - osteosclerosis of adjacent bones (zygoma and hard palate) - periosteal reaction - sinusitis |

*ocal medullary sclerosis with trabecular disorganisation and poor cortico-medullary differentiation

**Table 4.** Clinical and radiological staging of MRONJ (SICMF-SIPMO)[68]

| **Clinical-radiological staging of MRONJ** | |
| --- | --- |
| **Stage 1** | **FOCAL MRONJ**: where there is at least 1 minor clinical sign and *bone thickening* *on the CT, which is limited to the dento-alveolar* process* of the mandible or maxilla, with or without other early radiological signs.  **Minor clinical signs**: halitosis, odontogenic abscess, mandibular asymmetry, bone exposure, mucosal fistula, mucosal hyperaemia, lack of post-extraction alveolar mucosal repair, rapid onset tooth mobility, paresthesia/dysesthesia of the lips, purulent discharge, spontaneous sequestration of bone fragments, lockjaw, soft tissue swelling.  **CT signs***: trabecular thickening, focal medullary osteosclerosis*, with or without alveolar ridge and lamina dura thickening, post-extraction alveolus persistence, periodontal space widening.  **a. asymptomatic**  **b. symptomatic** (presence of pain and/or suppuration) |
| **Stage 2** | **DIFFUSE MRONJ**: where there is at least 1 minor clinical sign and *bone thickening on the CT scan also extending to the basal process* of the mandible or maxilla, with or without late radiological signs.  **Minor clinical signs**: as for Stage 1.  **CT signs**: *diffuse osteosclerosis*, with or without oro-antral and oro-nasal fistula, thickening of the alveolar canal, periosteal reaction, sequestration, sinusitis.  **a. asymptomatic**  **b. symptomatic** (presence of pain and/or suppuration) |
| **Stage 3** | **COMPLEX MRONJ:** as in Stage 2, in the presence of one or more of the following:  **Minor clinical signs:** extra-oral fistula, leakage of fluid from the nose, with or without altered occlusion  **CT signs:** muco-cutaneous fistula, pathological fracture, osteolysis extended to maxillary sinus, osteosclerosis of cheekbone and/or hard palate.  **a. asymptomatic**  **b. symptomatic** (presence of pain and/or suppuration) |

* The dento-alveolar region is defined as the anatomical bone structure providing skeletal support to the teeth. By definition, the dento-alveolar process terminates in a cranio-caudal position, just below the teeth roots.

**Figure 1:** Hypothesis of risk gradient for MRONJ in cancer patients (SICMF-SIPMO)[68]


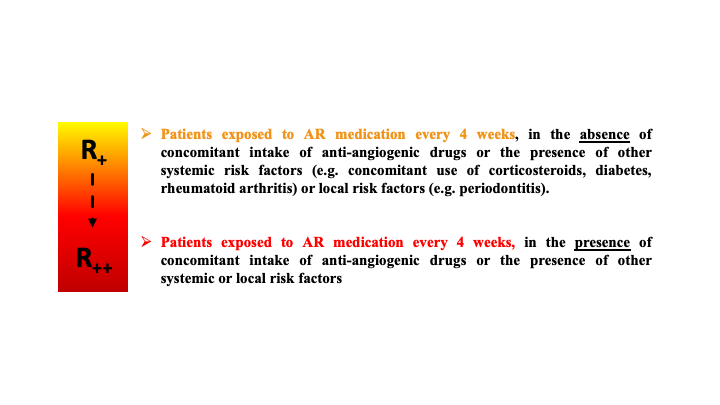


**N.B.** Cancer patients receiving hormonal therapy and ONJ-related drugs for the prevention of cancer treatment induced bone loss (CTIBL) are not considered within the risk gradient. The risk of ONJ in this group of patients is comparable to osteometabolic patients being treated with ONJ-related drugs, in the presence of other systemic and/or local risk factors.

**Figure 2. –** Hypothesis of risk gradient for MRONJ in osteometabolic patients (SICMF-SIPMO)[68]

**
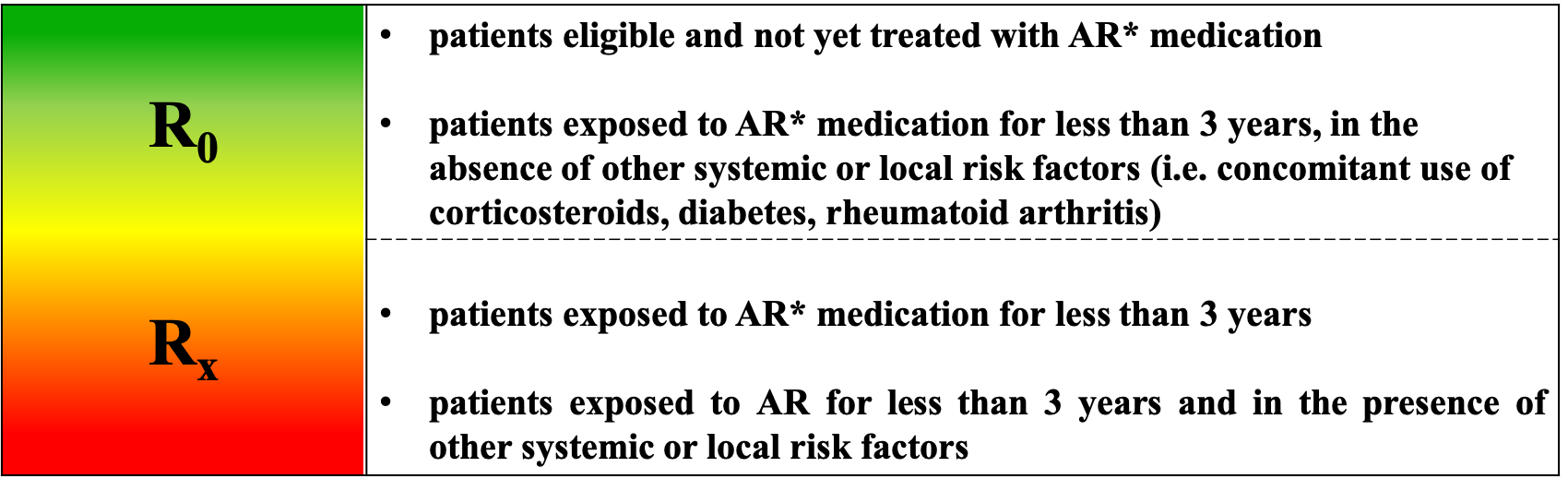
**

***** Antiresorptive (AR) medication for osteometabolic patients are: Denosumab (i.e. Prolia ®), BPs (*per o*, *im* or *ev*)

**N.B.** The risk of MRONJ in cancer patients receiving hormonal therapy and MRONJ-related drugs*, for the prevention of cancer treatment induced bone loss (CTIBL), is comparable to osteometabolic patients in treatment with ONJ-related drugs, with other systemic and/or local risk factors.

**Figure 3.** Diagnostic work-up of MRONJ (modified from SICMF-SIPMO)[68]
