# Supplementary material for: The preventive care of medication-related osteonecrosis of the jaw (MRONJ): a position paper by Italian experts for dental hygienists
Source: Support Care Cancer. 2022 Mar 16;30(8):6429–40. doi: 10.1007/s00520-022-06940-8 (PMC9213300; doi:10.1007/s00520-022-06940-8)
Supplement: Supplementary file 2 — Supplementary file2 (DOCX 63 KB) [file 520_2022_6940_MOESM2_ESM.docx]

**The preventive care of Medication-related Osteonecrosis of the Jaw (MRONJ): a Position Paper by Italian experts for dental hygienists**

**Authors**

Mauceri Rodolfo*^1,2,3^, Coniglio Rita^1^, Abbinante Antonia^4^, Carcieri Paola^5^, Tomassi Domenico^6^, Panzarella Vera^1^, Di Fede Olga^1^, Bertoldo Francesco^7^, Fusco Vittorio^8^, Bedogni Alberto^9^, Campisi Giuseppina^1^.

**Affiliations**

^1^ Department of Surgical, Oncological and Oral Sciences (Di.Chir.On.S.), University of Palermo, Palermo, Italy

^2^ Department of Biomedical and Dental Sciences, Morphological and Functional Images, University of Messina, Messina, Italy

^3^ Department of Dental Surgery, Faculty of Dental Surgery, University of Malta, Msida, Malta

^4^ Italian Dental Hygienists Association - AIDI, Aosta, Italy

^5^ Department of Surgical Sciences, Oral Medicine Section, CIR-Dental School, University of Turin, Turin, Italy - Oral prevention and community dentistry, CIR-Dental School, University of Turin, Turin, Italy

^6^ Catholic University of Rome, Rome, Italy - National Union Of Dental Hygienists – UNID, Rome, Italy

^7^ Department of Medicine, University of Verona, Verona, Italy.

^8^ Oncology Unit, Azienda Ospedaliera di Alessandria SS, Antonio e Biagio e Cesare Arrigo, Alessandria, Italy

^9^ Regional Center for Prevention, Diagnosis and Treatment of Medication and Radiation-Related Bone Diseases of the Head and Neck, University of Padua, Padua, Italy.

*Corresponding Author

Rodolfo Mauceri,

Sector of Oral Medicine, Department of Surgical, Oncological and Oral Sciences (Di.Chir.On.S),

University of Palermo, Palermo, Italy

Via L. Giuffrè 5, 90127

Palermo (PA), Italy

rodolfo.mauceri@unipa.it

**Appendix 2**

**Chronic inflammatory periodontitis and peri-implantitis as local risk factors for MRONJ**

*Health promotion* is the process of enabling people to take greater control of and improve their health[1]. The prevention of disease not only involves measures to preclude disease onset, such as a reduction in risk factors, but it also measures the impeding of the progress of an existing disease by reducing its consequences. Indeed, the aims of primary prevention are to prevent the outbreak of a disease, while the aims of secondary and tertiary prevention regard the arresting or delaying of existing diseases and their effects. The latter is effected through early diagnosis and appropriate treatment, or at delaying relapses and the transition to a chronic state.

Periodontitis is a chronic inflammation, which is caused by specific pathogens contained in dental plaque, leading to host/bacterial dysbiosis and resulting in: connective tissue destruction, bone resorption and tooth loss[2]. It is the sixth most common, chronic non-communicable disease in the world[3] and the main cause of tooth loss. In its various manifestations, periodontitis has a marked prevalence (over 40%) among individuals living in industrialized countries, while the most severe forms permanently involve more than 10% of the world's population[4, 5], with significant associated cosmetic, masticatory and psychological problems. Recognized risk factors for these diseases include: smoking, uncompensated diabetes, stress, obesity and a genetic predisposition[6].

Extensive data has demonstrated that periodontitis interacts with many systemic diseases, including cardiovascular disease and diabetes[7]. The hypothetical mechanisms underlying these interactions are based on a systemic inflammation caused by periodontitis. This involves the production of pro-inflammatory molecules and the release of bacterial toxins at a systemic level, which can alter and complicate the profile of existing chronic diseases[7]. Periodontitis can be characterized by means of a classification system, describing the stage and extent of the disease (Table 1). A given stage is defined by the severity of the disease and the complexity of case management, and it includes the extent and distribution of the disease (Table 1). The extent of the disease provides information regarding the patient's risk factors and rate of progression, and it should form the basis of individual treatment planning (Table 2)[8, 9].

The initial stage of periodontitis should be determined by using clinical attachment loss (CAL); if unavailable, radiographic bone loss (RBL) can be used. Information relating to tooth loss, which can be primarily attributed to periodontitis, where available, may alter stage definition. This is the case even in the absence of factors of complexity. The latter can shift the stage into a higher level, eg. a Cass II or III furcation would move the classification to a Stage III or IV, irrespective of the CAL. The distinction between Stage III and Stage IV is based primarily on factors of complexity. For example, a marked degree of tooth mobility and/or posterior bite collapse would indicate a Stage IV diagnosis. For any given case, only some - not all - of the factors of complexity may be present. However, a single complexity factor is generally sufficient to label the diagnosis into a higher stage. It should also be stressed that these case definitions are guidelines, to be applied using sound clinical judgement in arriving at the most accurate clinical diagnosis. CAL and RBL are still the main determinants regarding stage for post-treatment patients. If the treatment eliminates a complexity factor, which is capable of modifying the stage, the latter should not be downgraded to a lower stage as the complexity factor of the original stage should always be kept in mind in managing the maintenance phase.

The extent of periodontitis should be used as an indicator of the rate its progression; the main criteria are direct or indirect evidence of this progression. Where available, direct evidence is used; where absent, an indirect estimate can be made, using age-dependent bone loss at the most affected tooth or when presenting (radiographic bone loss expressed as a % of the root length divided by the patient’s age, RBL/age). The clinician should initially assume a Grade B disease and then ascertain the presence of specific evidence to move to Grade A or Grade C, where appropriate. Once the stage, based on evidence of progression, has been established, it can be modified according to the presence of risk factors.

In cases of patient with dental implants, it should be considered that mucositis and/or peri-implantitis may be present. The main clinical characteristic of peri-implant mucositis is bleeding on gentle probing. Other clinical signs of mucositis may be mild erythema, swelling and/or suppuration. The presence of mucositis can be evaluated through the presence of BoP, not excluding the possible presence of the other clinical signs[10]. Peri-implantitis is a plaque-associated pathological condition, occurring in tissues around dental implants, characterized by inflammation in the peri-implant mucosa and subsequent and progressive loss of supporting bone[10].

A diagnosis of peri-implantitis requires: the presence of bleeding and/or suppuration on gentle probing; increased probing depth, compared to previous examinations; the presence of bone loss beyond the crestal bone level, and changes resulting from initial bone remodeling. In the absence of previous examination data, a diagnosis of peri-implantitis can be based on the combination of: the presence of bleeding and/or suppuration on gentle probing; probing depths ≥6 mm; bone levels ≥3 mm, which is apical of the most coronal portion of the intraosseous part of the implant [10](Table 3).

As stated by Berglundh et al, these definitions should be viewed within a context of the absence of a generic implant and the presence of numerous implant designs with different surface characteristics, surgical and loading protocols. In order to plan for effective follow-up, safeguarding peri-implant and MRONJ health, the obtaining of baseline radiographic and probing measurements is recommended, following the placement of prosthetic restoration on dental implants.

**References**

1. World Health Organization (1986) Ottawa Charter for Health Promotion

2. Sanz M, Quirynen M (2005) Advances in the aetiology of periodontitis: Group A Consensus report of the 5th European Workshop in Periodontology. In: Journal of Clinical Periodontology. J Clin Periodontol, pp 54–56

3. Tonetti MS, Jepsen S, Jin L, Otomo-Corgel J (2017) Impact of the global burden of periodontal diseases on health, nutrition and wellbeing of mankind: A call for global action. J Clin Periodontol 44:456–462. https://doi.org/10.1111/jcpe.12732

4. Kassebaum NJ, Bernabé E, Dahiya M, et al (2014) Global burden of severe periodontitis in 1990-2010: A systematic review and meta-regression. J. Dent. Res. 93:1045–1053

5. Aimetti M, Perotto S, Castiglione A, et al (2015) Prevalence of periodontitis in an adult population from an urban area in North Italy: Findings from a cross-sectional population-based epidemiological survey. J Clin Periodontol 42:622–631. https://doi.org/10.1111/jcpe.12420

6. Genco RJ, Borgnakke WS (2013) Risk factors for periodontal disease. Periodontol 2000 62:59–94. https://doi.org/10.1111/j.1600-0757.2012.00457.x

7. Linden GJ, Lyons A, Scannapieco FA (2013) Periodontal systemic associations: review of the evidence. J Periodontol 84:S8–S19. https://doi.org/10.1902/jop.2013.1340010

8. Papapanou PN, Sanz M, Buduneli N, et al (2018) Periodontitis: Consensus report of workgroup 2 of the 2017 World Workshop on the Classification of Periodontal and Peri-Implant Diseases and Conditions

9. Tonetti MS, Greenwell H, Kornman KS (2018) Staging and grading of periodontitis: Framework and proposal of a new classification and case definition. J Periodontol 89:S159–S172. https://doi.org/10.1002/JPER.18-0006

10. Berglundh T, Armitage G, Araujo MG, et al (2018) Peri-implant diseases and conditions: Consensus report of workgroup 4 of the 2017 World Workshop on the Classification of Periodontal and Peri-Implant Diseases and Conditions. In: Journal of Clinical Periodontology. Blackwell Munksgaard, pp S286–S291

**Tables:**

**Table 1.** Criteria for defining stages of periodontitis (from Tonetti et al., 2018)[9]

| **Periodontitis stage** | | **Stage I** | **Stage II** | **Stage III** | **Stage IV** |
| --- | --- | --- | --- | --- | --- |
| **Severity** | **Interdental CAL at site of maximum loss** | 1 to 2 mm | 3 to 4 mm | ≥5 mm | ≥5 mm |
|  | **Radiographic bone loss** | Coronal third (<15%) | Coronal third (15% to 33%) | Extending to mid-third of root and beyond | Extending to mid-third of root and beyond |
|  | **Tooth loss** | No tooth loss due to periodontitis | | Tooth loss due to periodontitis of ≤ 4 teeth | Tooth loss due to periodontitis of ≥5 teeth |
| **Complexity** | **Local** |  |  | In addition to stage II complexity: | In addition to stage III complexity: |
|  |  | Maximum probing depth ≤4 mm | Maximum probing depth ≤5 mm | Probing depth ≥ 6 mm | Need for complex rehabilitation due to: |
|  |  | Mostly horizontal bone loss | Mostly horizontal bone loss | Vertical bone loss ≥ 3 mm | Masticatory dysfunction  Secondary occlusal trauma (tooth mobility degree ≥ 2)  Severe ridge defect  Bite collapse, drifting, flaring  Less than 20 remaining teeth (10 opposing pairs) |
|  |  |  |  | Furcation involvement Class II or III |  |
|  |  |  |  | Moderate ridge defect |  |
| **Extent and distribution** | **Add to stage as descriptor** | For each stage, describe extent as localized (<30% of teeth involved), generalized, or molar/incisor pattern | | | |

**Table 2.** Criteria for defining the degree of periodontitis (from Tonetti et al., 2018)[9]

| **Periodontitis grade** | | | **Grade A: slow rate of progression** | **Grade B: moderate rate of progression** | **Grade C: rapid rate of progression** |
| --- | --- | --- | --- | --- | --- |
| **Primary criteria** | Direct evidence of progression | Longitudinal data (radiographic bone loss or CAL) | Evidence of no loss over 5 years | <2 mm over 5 years | ≥2 mm over 5 years |
|  | Indirect evidence of progression | % bone loss/age | <0.25 | 0.25 to 1.0 | >1.0 |
|  |  | Case phenotype | Heavy biofilm deposits with low levels of destruction | Destruction commensurate with biofilm deposits | Destruction exceeds expectation, given biofilm deposits; specific clinical patterns suggestive of periods of rapid progression and/or early onset disease (e.g., molar/incisor pattern; lack of expected response to standard bacterial control therapies) |
| **Grade modifiers** | Risk factors | Smoking | Non-smoker | Smoker <10 cigarettes/day | Smoker ≥10 cigarettes/day |
|  |  | Diabetes | Normoglycemic / no diagnosis of diabetes | HbA1c <7.0% in patients with diabetes | HbA1c ≥7.0% in patients with diabetes |
| **Risk of systemic impact of periodontitis^a^** | Inflammatory burden | High sensitivity CRP (hsCRP) | <1 mg/L | Da 1 a 3 mg/L | >3 mg/L |
| **Biomarkers** | Indicators of CAL/bone loss | Saliva, gingival crevicular fluid, serum | ? | ? | ? |

^a^ This refers to an increased risk, in that periodontitis may be an inflammatory comorbidity for that patient. The CRP values provide a summary of the patient's overall systemic inflammation, which may be partly influenced by periodontitis. It may alternatively indicate an ‘unexplainable’ inflammation, which can assist the physician in their assessment. The grey colour of the cells in Table 2 refers to the requirement to substantiate this data with specific evidence. This item has been included in Table 2 to highlight the extent of the biology of periodontitis. In the future, it can be expected that it will be possible to integrate information regarding periodontitis in order to highlight the potential systemic impact of this disease on the specific case. The *question marks* in the last row indicate that specific biomarkers and related thresholds may be incorporated into the Table as soon as scientific evidence becomes available.

HbA1c, glycated haemoglobin; hsCRP, high-sensitivity C-reactive protein; PA, periapical; CAL, clinical attachment loss.

**Table 3.** Peri-Implant Disease and Conditions [10]

|  | **Peri-Implant Health** | **Peri-Implant Mucositis** | **Peri-Implantitis** |
| --- | --- | --- | --- |
| **Clinical characteristics** | Characterized by absence of erythema, bleeding on probing, swelling and suppuration. | Characterized by bleeding on gentle probing.  Erythema, swelling and/or suppuration may also be present. | A plaque-associated pathological condition, occurring in tissues around dental implants.  Characterized by inflammation in the peri-implant mucosa and subsequent progressive loss of supporting bone. |
| **Clinical differences between healthy peri-implant and periodontal tissues** | There are no visual differences between peri- implant and periodontal tissues.  The probing depths are usually greater at implant versus tooth sites.  The papillae at the interproximal sites of an implant may be shorter than the papillae at interproximal tooth sites. | An increase in probing depth is often observed in the presence of peri-implant mucositis due to swelling or decrease in probing resistance. | Peri-implantitis sites exhibit clinical signs of inflammation, bleeding on probing and/or suppuration, increased probing depths and/or recession of the mucosal margin in addition to radiographic bone loss compared to previous examinations.  At sites presenting with peri-implantitis, probing depth is correlated with bone loss and is, hence, an indicator for the severity of disease. |
| **Case definitions** | Diagnosis of peri-implant health requires:  • an absence of clinical signs of inflammation.  • an absence of bleeding and/or suppuration on gentle probing.  • no increase in probing depth compared to previous examinations.  • an absence of bone loss beyond crestal bone level changes, resulting from initial bone remodeling. | Diagnosis of peri-implant mucositis requires a:  presence of bleeding and/or suppuration on gentle probing with or without increased probing depth compared to previous examinations.  • an absence of bone loss beyond crestal bone level changes, resulting from initial bone remodeling. | Diagnosis of peri-implantitis requires^b^:  A presence of bleeding and/or suppuration on gentle probing.  • increased probing depth compared to previous examinations.  • a presence of bone loss beyond crestal bone level changes, resulting from initial bone remodeling.  In the absence of previous examination data, a diagnosis of peri-implantitis can be based on the combination of:  • the presence of bleeding and/or suppuration on gentle probing.  • probing depths of ≥6 mm and  • bone levels ≥3 mm, apical of the most coronal portion of the intraosseous part of the implant. |

^a^ It should be noted that visual signs of inflammation can vary and that peri-implant mucositis can exist around implants with varying levels of bone support.^b^ It should be noted that visual signs of inflammation can vary and that recession of the mucosal margin should be considered in evaluating probing depth.
